# A Novel Prognostic Signature for Survival Prediction and Immune Implication Based on SARS-CoV-2–Related Genes in Kidney Renal Clear Cell Carcinoma

**DOI:** 10.3389/fbioe.2021.744659

**Published:** 2022-01-24

**Authors:** Yongbiao Huang, Sheng Chen, Lingyan Xiao, Wan Qin, Long Li, Yali Wang, Li Ma, Xianglin Yuan

**Affiliations:** ^1^ Department of Oncology, Tongji Hospital, Tongji Medical College, Huazhong University of Science and Technology, Wuhan, China; ^2^ Department of Hepatobiliary Surgery, Affiliated Hospital of Hebei University, Baoding, China

**Keywords:** kidney renal clear cell carcinoma, COVID-19, SARS-CoV-2, prognostic signature, nomogram

## Abstract

Kidney renal clear cell carcinoma (KIRC) is a common aggressive malignancy of the urinary system. COVID-19, a highly infectious and severe disease caused by SARS-CoV-2, has become a significant challenge for global public health. Cancer patients have been reported to be more vulnerable to SARS-CoV-2 infection and have a higher risk for serious complications than the general population. However, the correlation between KIRC and COVID-19 remains incompletely elucidated. In this study, we comprehensively investigated the expression and prognostic significance of 333 SARS-CoV-2 infection–related genes in KIRC using the TCGA dataset and identified 31 SARS-CoV-2–related differently expressed genes between KIRC and normal renal tissues. Based on these genes, we constructed and validated a 5-gene prognostic signature (including *ACADM*, *CENPF*, *KDELC1*, *PLOD2*, and *TRMT1*) to distinguish low- and high-risk KIRC patients of poor survival in TCGA and E-MTAB-1980 cohorts. Gene set enrichment analysis (GSEA) showed that some inflammatory/immune-related pathways were significantly enriched in the high-risk group. The ESTIMATE analysis indicated that patients in the high-risk group had higher stromal and immune cell scores, therefore lower tumor purity. Moreover, they presented higher proportions of macrophages M0, regulatory T cells (Tregs), and T follicular helper cells and higher expression of immune checkpoints CTLA-4, LAG-3, TIGIT, and PDCD1 than low-risk patients. Besides, we also developed a nomogram to expand clinical applicability, which exhibits excellent predictive accuracy for survival. In conclusion, we identified a novel prognostic signature and nomogram based on SARS-CoV-2–related genes as reliable prognostic predictors for KIRC patients and provided potential therapeutic targets for KIRC and COVID-19.

## Introduction

Renal cell carcinoma (RCC) has been considered as the third most common malignancy of the urinary system in adults, with high morbidity and mortality (Cohen and McGovern, 2005; Rini et al., 2009). More than 300,000 new cases are diagnosed, and over 100,000 patients die from it per year worldwide; it has gradually become a global public health burden (Siegel et al., 2020). Kidney renal clear cell carcinoma (KIRC) is the most frequent histopathologic subtype of RCC, accounting for approximately 75% of all primary RCCs (Jonasch et al., 2014; Moch et al., 2016). Despite recent improvements in multiple therapeutic methods, including radical resection, chemoradiotherapy, targeted therapy, and immunotherapy, the prognosis for most KIRC patients remains poor because of the high risk of local recurrence and distant metastasis (Barata and Rini, 2017; Choueiri and Motzer, 2017). The recurrence rate of KIRC after initial curative nephrectomy varies from 20 to 30% within 5 years, and the 5-year overall survival rate of metastatic KIRC patients is less than 10%. Up to 30% of KIRC patients already had metastases at the first time of diagnosis and missed the optimal treatment opportunity (Cohen and McGovern, 2005; Rini et al., 2009). Thus, it is imperative to explore novel reliable biomarkers for early diagnosis and prognosis prediction and provide prognostic indicators and treatment targets for KIRC.

Severe acute respiratory syndrome coronavirus 2 (SARS-CoV-2), a new coronavirus infecting humans, was first identified in Wuhan, China, in December 2019 and spread rapidly around the world and caused a global coronavirus disease 2019 (COVID-19) pandemic within several months (Tian et al., 2020; Wang et al., 2020). As of December 26, 2021, more than 279 million confirmed cases of SARS-CoV-2 infection and 5.39 million COVID-19–related deaths have been reported worldwide, based on the latest statistics from Johns Hopkins University (https://coronavirus.jhu.edu/map.html). Epidemiological studies so far have demonstrated that patients diagnosed with cancer have a higher risk of SARS-CoV-2 infection and resulted in serious complications and poor prognosis, especially in patients undergoing anticancer treatments (chemotherapy, surgery, or radiotherapy) and patients with hematologic malignancies (Dai et al., 2020; Liang et al., 2020; Wang and Zhang, 2020; Yu et al., 2020).

A recent study revealed that the SARS-CoV2 receptors or auxiliary proteins ACE2, ANPEP, ENPEP, and DPP4 are highly expressed in renal tumor, especially in KIRC. Furthermore, these SARS-CoV2 receptors are closely correlated with immune infiltrates and immune response in KIRC (Mihalopoulos et al., 2020; Tripathi et al., 2020). However, besides the several aforementioned SARS-CoV-2–related proteins, more than 300 SARS-CoV-2–interacting human proteins have been identified (Gordon et al., 2020), and the correlations between their expression, prognosis, and immune cell infiltration in KIRC tissues have not been elucidated. Thus, our study was carried out to explore the expression levels, prognostic value, and immune perspective of these SARS-CoV-2–related proteins in KIRC and further construct a SARS-CoV-2–related prognostic signature for KIRC. This study revealed the important roles of SARS-CoV-2–interacting proteins in KIRC and illustrated the susceptibility of KIRC patients to SARS-CoV-2 infection and the underlying mechanism of COVID-19 in KIRC patients.

## Materials and Methods

### Data Collection and Processing

The mRNA expression profile data and corresponding clinical information of 539 KIRC samples and 72 adjacent non-tumor samples were acquired from TCGA website, and the 333 SARS-CoV-2 infection–related proteins (Supplementary Table S1) were summarized in the Human Protein Atlas (HPA) database. Then, we utilized the “limma” R package to identify the differentially expressed genes (DEGs) between KIRC tissues and adjacent normal tissues in the TCGA-KIRC cohort and calculated the intersections between DEGs and SARS-CoV-2–related genes using an online tool Venny2.1. The threshold for significance was set at a false discovery rate (FDR) < 0.05 and |log2fold change (FC)|  ≥ 1. Besides, the interaction network for overlapping SARS-CoV-2–related DEGs was generated by the “GeneMANIA” online database to predict their function (Franz et al., 2018).

### Construction of a Prognostic SARS-CoV-2–Related Gene Signature

Based on SARS-CoV-2–related DEGs, the “survival” R package was used to construct a prognostic model. The KIRC patients in the SARS-CoV-2–related gene signature building and validation need to meet the following inclusion criteria: 1) complete SARS-CoV-2–related DEG expression profile; 2) complete clinical data (including age at diagnosis, gender, TNM stage, histological grade, survival status, and survival time); 3) the follow-up or survival time must be longer than 30 days. After that, 501 KIRC patients were included for the subsequent signature construction in total. We randomly divided the TCGA-KIRC dataset (*n* = 501) into a training cohort (*n* = 252) and a testing cohort (*n* = 249) to construct and validate the prognostic signature, respectively.

Next, we performed a univariate Cox regression analysis to evaluate the prognostic value of SARS-CoV-2–related DEGs in the training cohort and screen genes significantly associated with the overall survival (*p* < 0.01). Subsequently, the multiple stepwise Cox regression analysis was applied to develop an optimal predictive model for the overall survival on the preliminary-screened prognosis-related genes. According to the smallest Akaike information criterion (AIC) value, the regression coefficients of each gene in this signature were generated (Song et al., 2020). The risk score for each KIRC patient was calculated as follows: $\mathrm{Risk score=}\overset{n}{\underset{i}{\sum }}\mathrm{(Expression of gene i \times Coefficient i)}$ . The patients were ordered according to their risk scores and categorized into the high-risk and low-risk groups based on the median risk score.

### Evaluating the Prediction Power of the Prognostic Signature and Validation

The Kaplan–Meier method was used to plot survival curves and compare the differences of survival between high- and low-risk groups in the training cohort (*n* = 252). Additionally, the time-dependent receiver operating characteristic (ROC) curve analyses were implemented to assess the predictive capability for 1-, 3-, and 5-year OS of the prognostic signature and other clinical parameters by using the “survivalROC” R package. We also performed univariate and multivariate analyses with Cox regression to estimate the effect of the gene signature together with other clinical characteristics on survival (Long et al., 2019).

Meanwhile, the robustness and accuracy of the gene signature were further validated in the testing cohort and the entire TCGA-KIRC cohort. Besides, the E-MTAB-1980 dataset included 101 KIRC patients with reliable prognostic information which was downloaded from the ArrayExpress database for external validation (Sato et al., 2013).

### Expression and Prognostic Significance Verifying

The online database GEPIA was utilized to confirm the differential mRNA expression of the signature constituent SARS-CoV-2–related genes based on GTEx and TCGA datasets, and the protein expression was detected in the Human Protein Atlas (HPA) database (Tang et al., 2019; Huang et al., 2020). The association of the signature constituent genes with OS was further verified in the Kaplan–Meier Plotter website, and the genetic alteration analyses were performed through the cBioportal database (Gao et al., 2013; Nagy et al., 2021). Besides, the relationships between the mRNA expression levels of risk signature genes and six immune cells (B cells, CD4^+^ T cells, CD8^+^ T cells, neutrophils, macrophages, and Dendritic cells) infiltration were investigated by using the TIMER database (Li et al., 2017).

### PCA and Gene Set Enrichment Analysis

PCA scatter plots were generated to profile the distribution patterns of KIRC patients in two groups *via* using the “scatterplot3d” R package. Gene set enrichment analysis (GSEA) was achieved by GSEA v4.0.3 software to detect different functional phenotypes between the high- and low-risk groups based on GO Biological Process ontology gene sets. FDR <0.25 and a nominal *p*-value < 0.05 were considered to be significant.

### Immune Infiltration Analysis in the Entire TCGA-KIRC Cohort

We used the “CIBERSORT” R package to analyze the profiles of 22 tumor-infiltrating immune cells and quantify the relative proportions in the entire KIRC cohort (*p* < 0.05). Then, we further analyzed the correlations between the 22 types of immune cells (Newman et al., 2015). The “ESTIMATE” algorithm was also applied to calculate the immune and stromal scores and infer the tumor purity of each KIRC sample using gene expression data (Yoshihara et al., 2013). In addition, the correlations between the risk score signature and five immune checkpoints (CTLA-4, LAG-3, TIGIT, TIM-3, and PDCD1) were determined by the Pearson correlation test, and their expression levels were compared between high- and low-risk patients.

### Developing and Validating a Predictive Nomogram

The clinical parameters including age, gender, TNM stage, histological grade, and the risk score signature were included in the development of the predictive nomogram to predict the overall survival of KIRC patients in the training test *via* the “rms” R package (Balachandran et al., 2015). The training test was split into two groups: high- and low-score groups, based on the total points of the nomogram. Then, we generated the Kaplan–Meier survival curve, ROC curve, and calibration plots of 1,3,5-OS to assess the predictive performance of this prognostic nomogram. Additionally, the good prediction performance of the nomogram was also further validated in the testing set, entire KIRC cohort, and E-MTAB-1980 dataset.

### Statistical Analysis

All statistical analyses were conducted by R software 3.6.3. The survival comparisons were carried out through the Kaplan–Meier method with the log-rank test. The *p* < 0.05 was considered to be statistically significant, unless otherwise stated.

## Results

**FIGURE 1:**
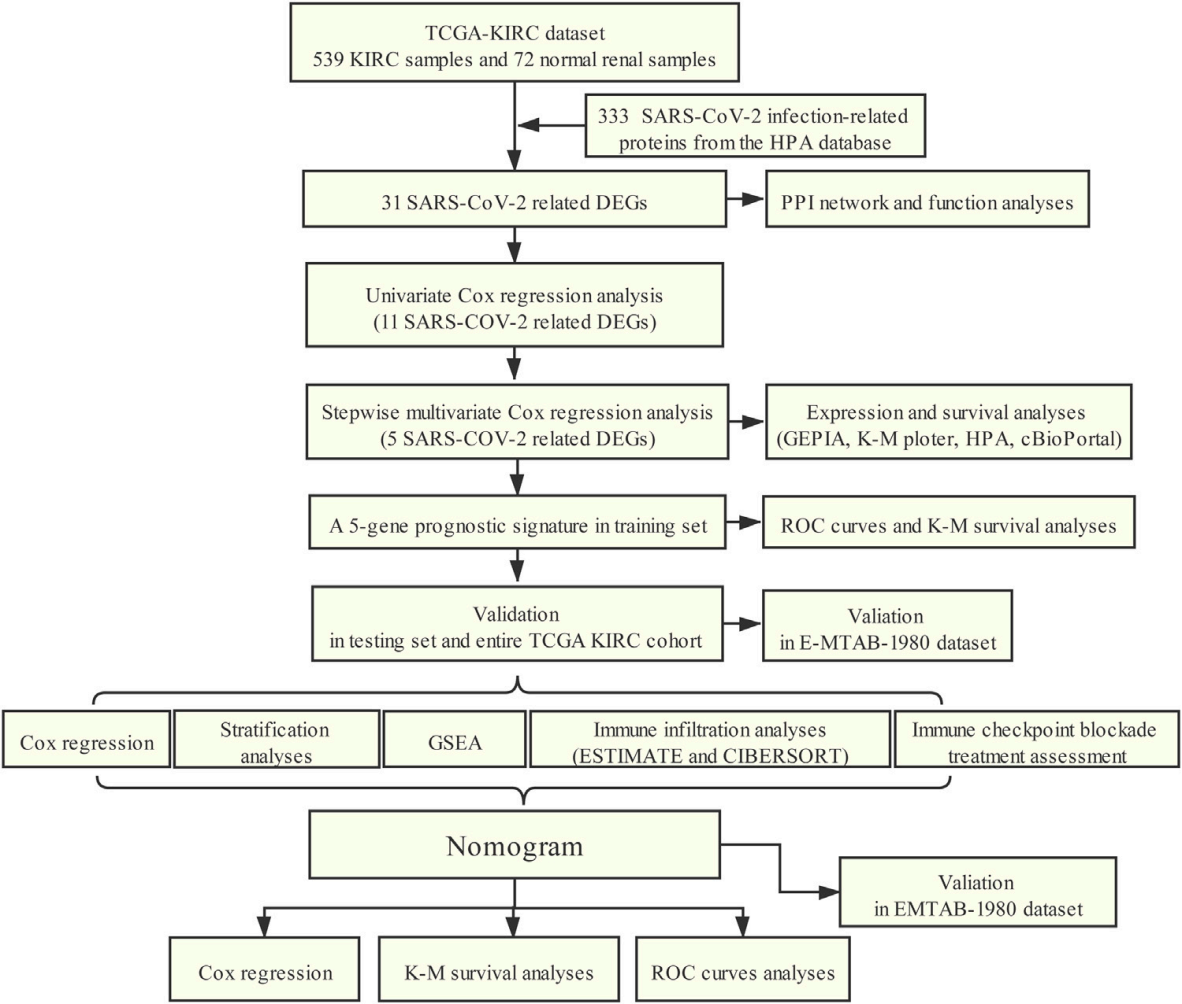
Workflow of this study.

The flowchart of our study was presented in Figure 1.

### Identifying DEGs and SARS-CoV-2–Related DEGs in the TCGA-KIRC Dataset

**FIGURE 2:**
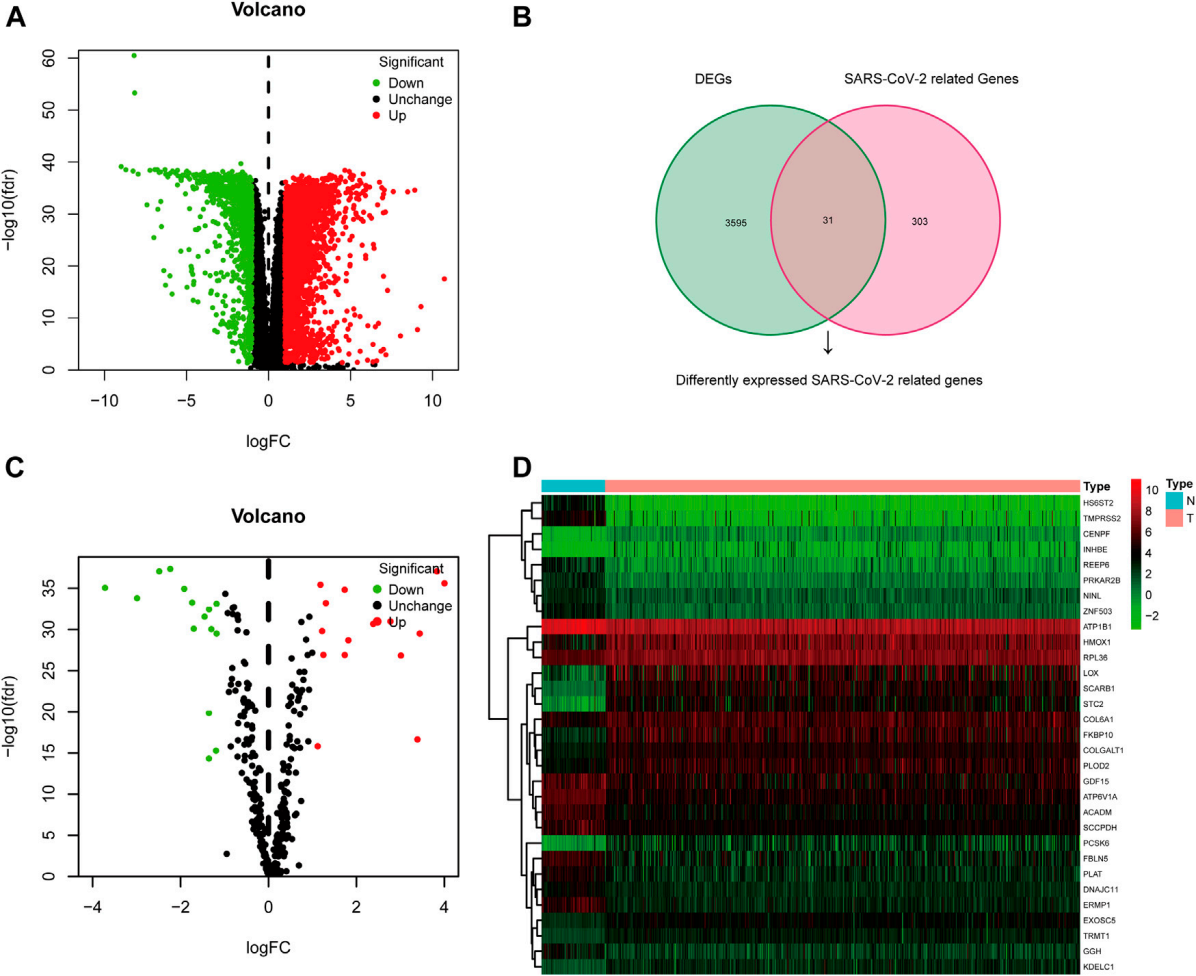
Differentially expressed SARS-CoV-2–related genes in KIRC. **(A)** Volcano plot of all DEGs; **(B)** Venn diagram to identify SARS-CoV-2–related DEGs; **(C)** Volcano plot of 31 SARS-CoV-2–related DEGs; **(D)** Heat map of 31 SARS-CoV-2–related DEGs.

The TCGA-KIRC dataset contains RNA sequencing data of 539 tumors and 72 adjacent normal tissues, and a total of 3,626 DEGs (2,513 upregulated and 1,113 downregulated) were identified between KIRC and normal kidney tissues (Figure 2A). We obtained the SARS-CoV-2–related gene list which contains 333 genes from the HPA online database and acquired 31 overlapped genes (SARS-CoV-2 related DEGs) including 15 upregulated and 16 downregulated between all DEGs and SARS-CoV-2–related genes by applying the Venn diagram (Figure 2B). The volcano plot and heatmap showing 31 SARS-CoV-2–related DEGs in Figures 2C,D. Moreover, the protein–protein interaction (PPI) network of SARS-CoV-2–related DEGs constructed using GeneMANIA uncovered these genes were primarily responsible for extracellular matrix and structure organization, exosome, and exonucleolytic nuclear-transcribed mRNA catabolic process, and deadenylation-dependent decay (Supplementary Figure S1).

### Constructing the SARS-CoV-2–Related Gene Signature in the Training Set

In total, 501 KIRC patients were eligible for our inclusion criteria and included in the risk score model construction and validation. All of them, 252 patients were randomized to the training set for model construction, and 249 were randomized to the training set for model validation. The detailed clinical information of these patients is summarized in Table 1. Through univariate Cox regression analysis, we detected 11 SARS-CoV-2–related DEGs significantly associated with prognosis (*p* < 0.01) in the training set (Supplementary Figure S2A). Subsequently, the stepwise multivariate Cox regression analysis was performed based on above 11 prognostic SARS-CoV-2–related genes, and a 5-gene prognostic signature was constructed in the training set (Supplementary Figure S2B; Supplementary Table S2). The detailed description about Covid-19 bait, tissue specificity, blood specificity, and subcellular location of the five hub SARS-CoV-2–related genes (*ACADM*, *CENPF*, *KDELC1*, *PLOD2*, and *TRMT1*) in this predictive model is listed in Supplementary Table S3. Among these five genes, only downregulated *ACADM* was the protective factor with Cox HR < 1, and the other upregulated *CENPF*, *KDELC1*, *PLOD2*, and *TRMT1* were unfavorable factors with Cox HR > 1. According to the expression and multivariate Cox regression coefficients of these five genes, we can calculate the risk score of each KIRC patient as follows: Risk score = (−0.4955 * ACADM Exp) + (0.3805 * CENPF Exp) + (0.2904 * KDELC1 Exp) + (0.2048 * PLOD2 Exp) + (0.4925 * TRMT1 Exp).

**TABLE 1:** Clinical characteristics of 501 KIRC patients involved in the 5-gene prognostic signature construction and validation.

Characteristics		Entire TCGA-KIRC cohort	Detailed data	
		(*N* = 501)	Training cohort (*N* = 252)	Testing cohort (*N* = 249)
Status				
	Dead	167 (33.3%)	80 (31.7%)	87 (34.9%)
	Alive	334 (66.7%)	172 (68.3%)	162 (65.1%)
Gender				
	Male	330 (65.9%)	162 (64.3%)	168 (67.5%)
	Female	171 (34.1%)	90 (35.7%)	81 (32.5%)
Age at diagnosis (years)				
	≤65	332 (66.3%)	172 (68.3%)	160 (64.3%)
	>65	169 (33.7%)	80 (31.7%)	89 (35.7%)
Histological grade				
	G1	11 (2.2%)	7 (2.8%)	4 (1.6%)
	G2	217 (43.3%)	104 (41.2%)	113 (45.4%)
	G3	201 (40.1%)	107 (42.5%)	94 (37.7%)
	G4	72 (14.4%)	34 (13.5%)	38 (15.3%)
TNM stage				
	I	250 (49.9%)	128 (50.8%)	122 (49.0%)
	II	53 (10.6%)	24 (9.5%)	29 (11.6%)
	III	116 (23.1%)	59 (23.4%)	57 (22.9%)
	IV	82 (16.4%)	41 (16.3%)	41 (16.4%)

**FIGURE 3:**
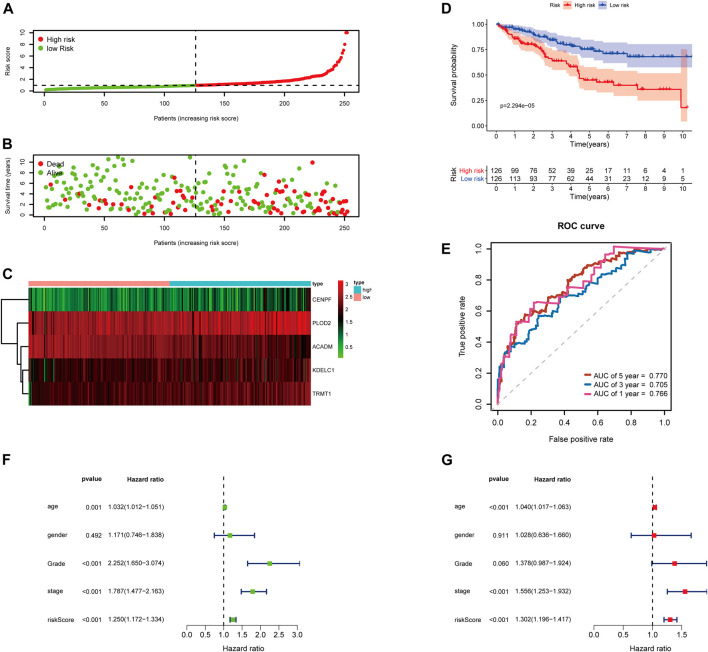
Risk score analysis of the 5-gene signature based on SARS-CoV-2–related genes in the training set. **(A–C)** Distribution of risk scores, OS status, and gene expression of model genes; **(D)** Kaplan–Meier curves of the high- and low-risk subgroup patients; **(E)** Time-dependent ROC curves for predicting 1-, 3-, and 5-year OS; **(F)** Univariate and **(G)** multivariate Cox regression analyses in the training set.

Then, the 252 KIRC patients in the training set were separated into low-risk (*n* = 126) or high-risk (*n* = 126) subgroups, using the median of the 5-gene signature risk scores as the cut-off (Figure 3A). The scatter plot for survival time and survival status distribution of each patient in the training cohort is illustrated in Figure 3B; patients with high risk usually had a higher likelihood of death earlier than those with low risk. Furthermore, the expression profile of the five genes in this signature was displayed as a heat map (Figure 3C). The Kaplan–Meier survival curves of two groups demonstrated that high-risk patients had a significant shorter survival time than those patients in the low-risk group, with *p* < 0.001 (Figure 3D). Moreover, the area under the curve (AUC) values of time-dependent ROC curves at 1-, 3-, and 5-years reached 0.766, 0.705, and 0.770, respectively; the results indicated that our prognostic signature had an excellent predictive ability (Figure 3E). Besides, the multivariate Cox regression analyses of the risk score signature and other clinical characteristics (age, gender, grade, and stage) were performed in the training set, and the risk score signature (HR = 1.302, 95% CI = 1.196–1.417, *p* < 0.001) was considered as an independent prognostic indicator for OS (Figures 3F,G; Supplementary Table S4).

### Validating the 5-Gene Risk Score Signature in the Testing Set and Entire TCGA-KIRC Cohort

**FIGURE 4:**
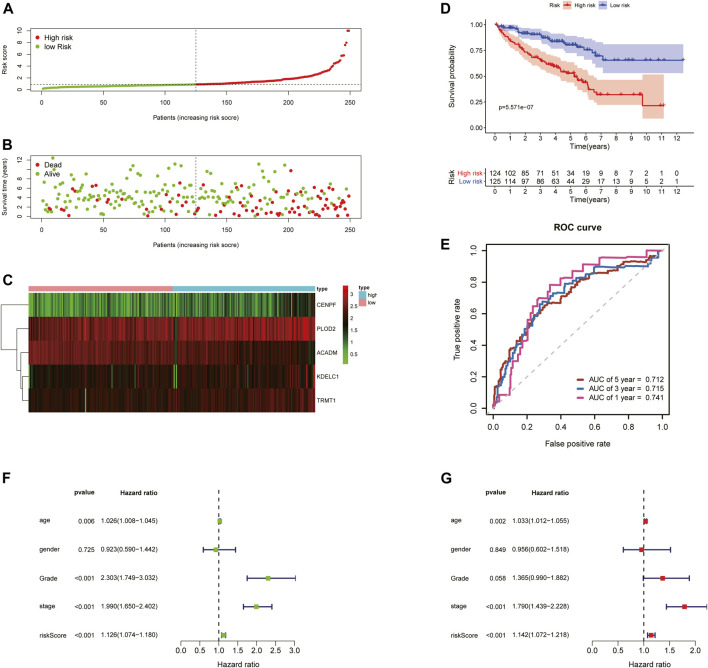
Risk score analysis of the 5-gene signature based on SARS-CoV-2–related genes in the testing set. **(A–C)** Distribution of risk scores, OS status, and gene expression of model genes; **(D)** Kaplan–Meier curves of the high- and low-risk subgroup patients; **(E)** Time-dependent ROC curves for predicting 1-, 3-, and 5-year OS; **(F)** Univariate and **(G)** multivariate Cox regression analyses in the testing set.

**FIGURE 5:**
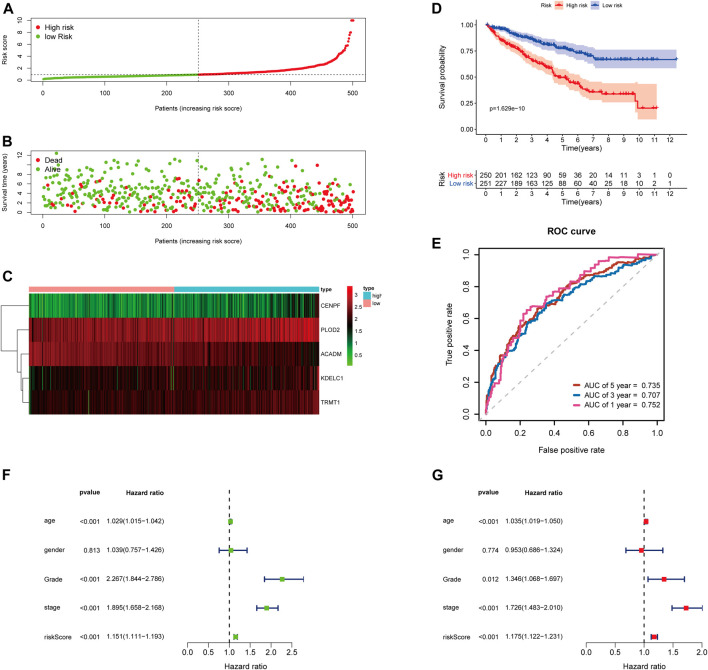
Risk score analysis of the 5-gene signature based on SARS-CoV-2–related genes in the entire TCGA-KIRC cohort. **(A–C)** Distribution of risk scores, OS status, and gene expression of model genes; **(D)** Kaplan–Meier curves of the high- and low-risk subgroup patients; **(E)** Time-dependent ROC curves for predicting 1-, 3-, and 5-year OS; **(F)** Univariate and **(G)** multivariate Cox regression analyses in the entire TCGA-KIRC cohort.

To further examine the predictive ability of this risk score model, we also calculated the risk scores of the KIRC patients in the testing cohort (*n* = 249) using the same formula (Figure 4). The Kaplan–Meier survival analysis for two groups demonstrated that high-risk patients had a significant poor prognosis than patients with low risk, with *p* < 0.001 (Figure 4D). And, the AUC values of time-dependent ROC curves at 1-, 3-, and 5-years reached 0.741, 0.715, and 0.712, respectively (Figure 4E). Besides, the results of multivariate Cox regression analyses revealed that the risk score signature (HR = 1.142, 95% CI = 1.072–1.218, *p* < 0.001) was an independent prognostic indicator for OS in the testing cohort (Figures 4F,G; Supplementary Table S4). In addition, we also obtained the similar results in the entire TCGA-KIRC cohort (*n* = 501) (Figure 5).

### Verification of Expression Levels and Prognostic Significance of the Five Genes

**FIGURE 6:**
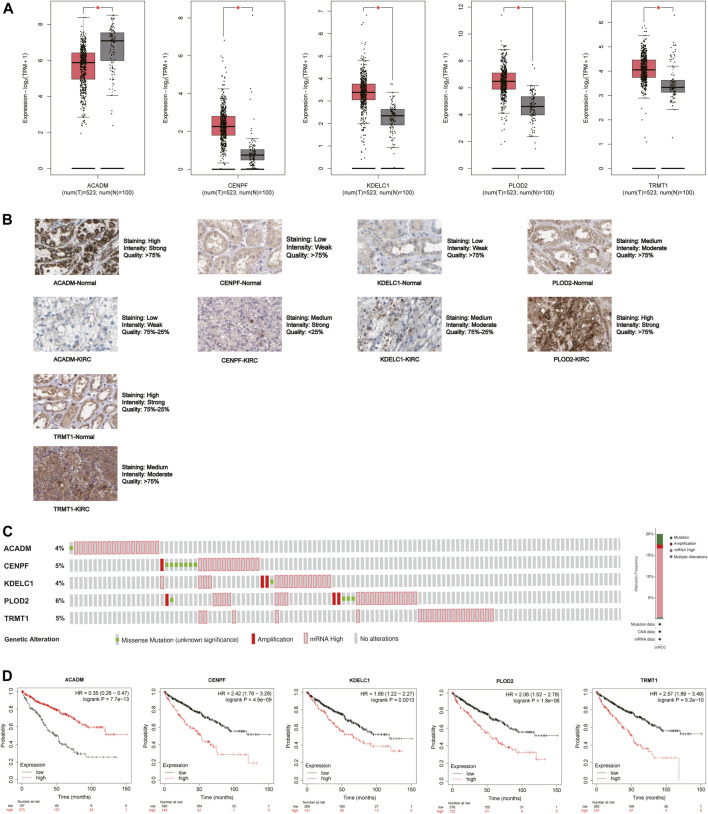
Expression and prognostic value of the five model genes (*ACADM*, *CENPF*, *KDELC1*, *PLOD2*, and *TRMT1*) in KIRC. **(A)** mRNA expression level between KIRC tissues and normal renal tissues in GEPIA; the cutoff of |log2FC| was set to 0.5, and the *p*-value was 0.05; **(B)** Protein expression level detected by immunohistochemistry in HPA; **(C)** Alteration frequency of the five hub SARS-CoV-2–related genes in 446 KIRC patients; **(D)** Prognostic value of five hub SARS-CoV-2–related genes in KIRC by the Kaplan Meier–plotter.

The mRNA and protein expression levels of the five hub genes (*ACADM*, *CENPF*, *KDELC1*, *PLOD2*, and *TRMT1*) were validated in GEPIA and HPA databases. The mRNA expression level of *ACADM* was significantly reduced in KIRC tissues, while those of *CENPF*, *KDELC1*, *PLOD2*, and *TRMT1* were significantly increased in KIRC tissues, which was determined by GEPIA (Figure 6A). The protein expression levels were detected through immunohistochemistry analyses in the HPA database, and the immunohistochemistry results further confirmed their expression difference between normal kidney tissues and KIRC tissues (Figure 6B). The cBioPortal online database includes 446 KIRC samples with complete genetic alteration data, 89 (20%) of them with alteration in the five genes, and the mRNA upregulation was the most frequent alteration type (Figure 6C). Besides, the KM survival curves of the five SARS-CoV-2–related genes were generated by the Kaplan–Meier plotter (splitting the patients by the best cut-off) to determine their relationship with OS. The results showed that a higher expression of *ACADM* (*p* = 7.7e-13) in KIRC predicted better OS, but instead the KIRC patients with higher expression levels of *CENPF* (*p* = 4.9e-09), *KDELC1* (*p* = 0.0013), *PLOD2* (*p* = 1.8e-06) and *TRMT1* (*p* = 5.2e-10) exhibited worse OS (Figure 6D). Moreover, it seems that these genes and their somatic copy number alteration were associated with the infiltration of B cells, CD4^+^ T cells, CD8^+^ T cells, neutrophils, macrophages, and dendritic cells in KIRC (Supplementary Figure S3). The correlation between the expression of the five genes is presented in Supplementary Figure S4; there was the greatest positive correlation between *KDELC1* and *PLOD2* (r = 0.39) and the greatest negative correlation between *ACADM* and *TRMT1* (r = −0.45).

### Stratification Analyses According to the Different Clinical Characteristics in the Entire TCGA-KIRC Cohort

**FIGURE 7:**
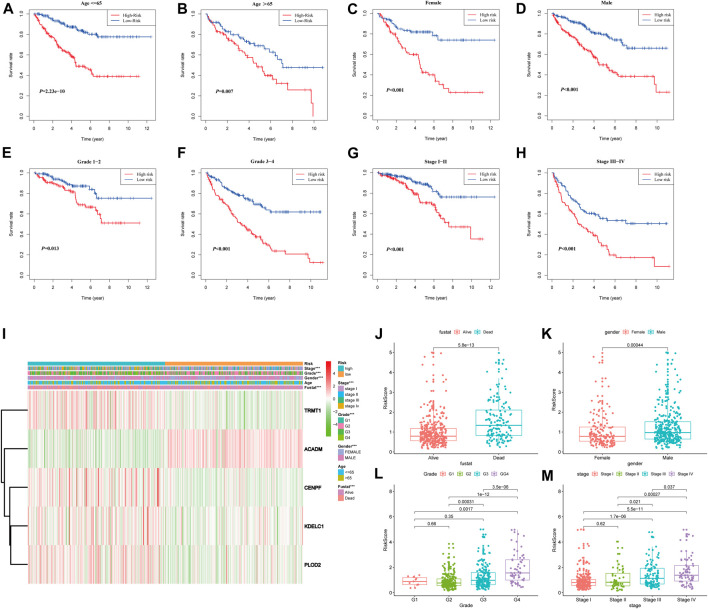
Stratified analyses based on the prognostic signature in the entire TCGA-KIRC cohort; **(A–H)** Kaplan–Meier survival analyses in different clinical characteristic subgroups; **(I)** Gene expression and clinicopathologic feature distribution of low- and high-risk groups; **(J–M)** Risk scores of patients stratified by survival status, gender, grade, and stage.

To further confirm the prognostic potential of the SARS-CoV-2–related gene signature in KIRC with different clinicopathological factors, we conducted series of subgroup survival analyses based on age, gender, grade, and stage in the entire TCGA-KIRC cohort. Survival curves revealed that patients in the high-risk group had a worse outcome compared to those in the low-risk group among different age (≤65 and >65), gender (male and female), grade (G1, G2, G3, and G4), and stage (I, II, III, and IV) subgroups (Figures 7A–H). The clinical characteristics of each patient in the entire TCGA-KIRC cohort and their corresponding risk score are summarized in Figure 7I, including the five model gene expression profiles. We also found that dead patients were more likely to have higher risk scores than alive patients (*p* < 0.001), suggesting that a higher risk score predicts a poorer prognosis (Figure 7J). For males, the risk scores were significantly higher than females (*p* < 0.001) (Figure 7K). Furthermore, the risk score was particularly relevant to the tumor malignant phenotype; patients with a higher tumor stage and historical grade usually had higher risk scores, which demonstrated that the prognostic signature could predict the progression of KIRC (Figures 7L,M).

### PCA and GSEA Analyses

**FIGURE 8:**
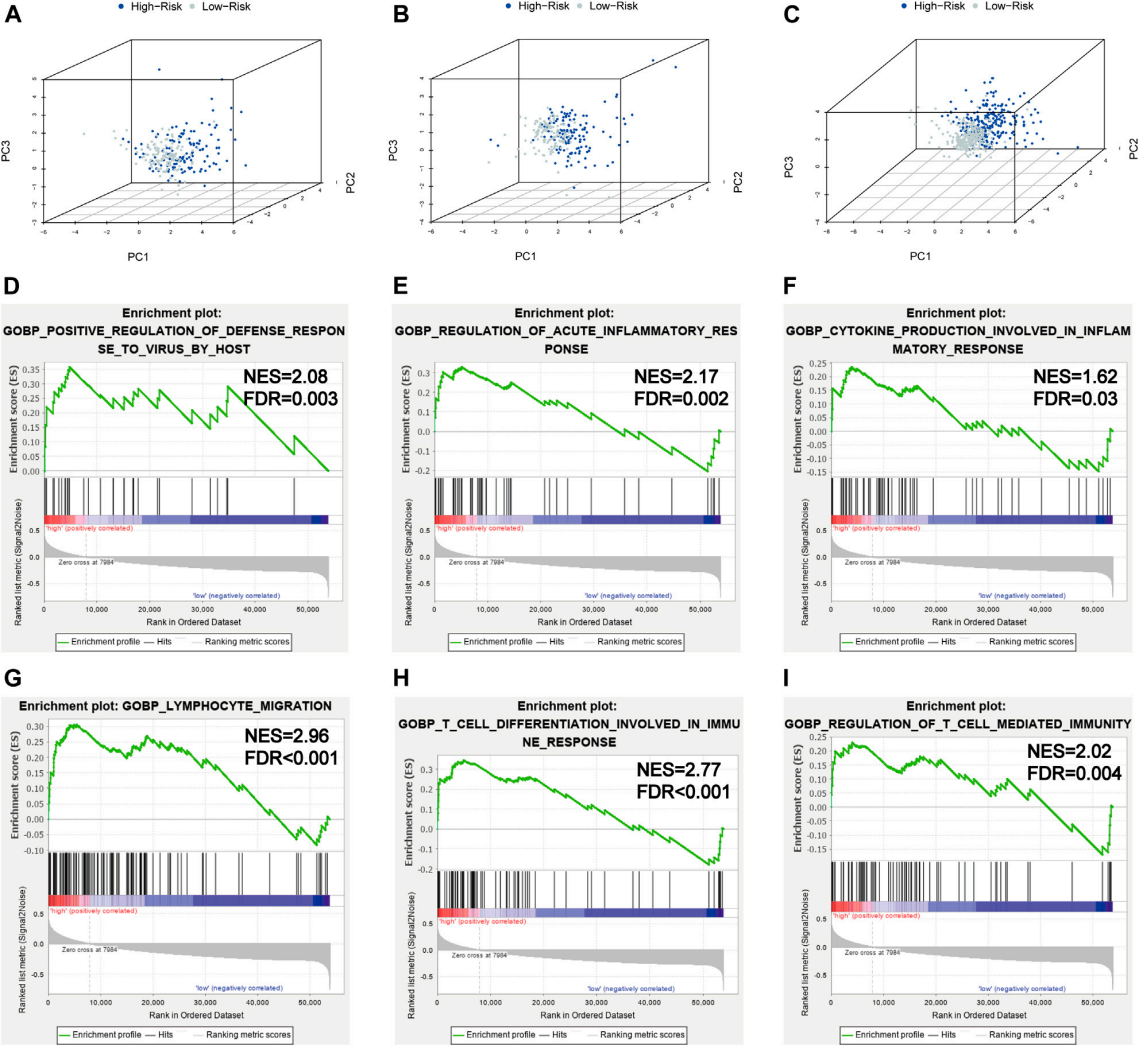
PCA and GSEA analyses. **(A–C)** PCA plots of the training set, testing set, and entire TCGA-KIRC cohort; **(D–I)** GSEA analyses for the biological processes in the entire TCGA-KIRC cohort.

In order to investigate the distribution patterns of patients in high- and low-risk groups, according to the expression of genes in the signature, we performed PCA analyses in the training cohort, testing cohort, and entire KIRC cohort. The results in the three cohorts all showed that patients from different risk groups were distributed in two distinct directions (Figures 8A–C).

Viral infection is associated with inflammation; SARS-CoV-2 infection could induce severe inflammatory responses and cytokine storms, contributing to serious events. To further illuminate the underlying biological processes related to the risk score signature, GSEA was implemented between high- and low-risk groups in the entire TCGA-KIRC cohort. We found some antiviral defense and inflammatory/immune-related processes were active in patients with high risks (Figures 8D–I), such as positive regulation of the defense response to the virus by the host (NES = 2.08, FDR = 0.003), regulation of the acute inflammatory response (NES = 2.17, FDR = 0.002), cytokine production involved in the inflammatory response (NES = 1.62, FDR = 0.03), lymphocyte migration (NES = 2.96, FDR < 0.001), T-cell differentiation involved in the immune response (NES = 2.77, FDR < 0.001), and regulation of T-cell–mediated immunity (NES = 2.02, FDR = 0.004). Taken together, the 5-gene risk score signature based on SARS-CoV-2–related genes could reflect the immune status and deduct the immune landscapes of KIRC patients, and high-risk patients were more likely to have a strong inflammatory response to SARS-CoV-2.

### The Immune Infiltration Landscapes Between the High- and Low-Risk Groups

**FIGURE 9:**
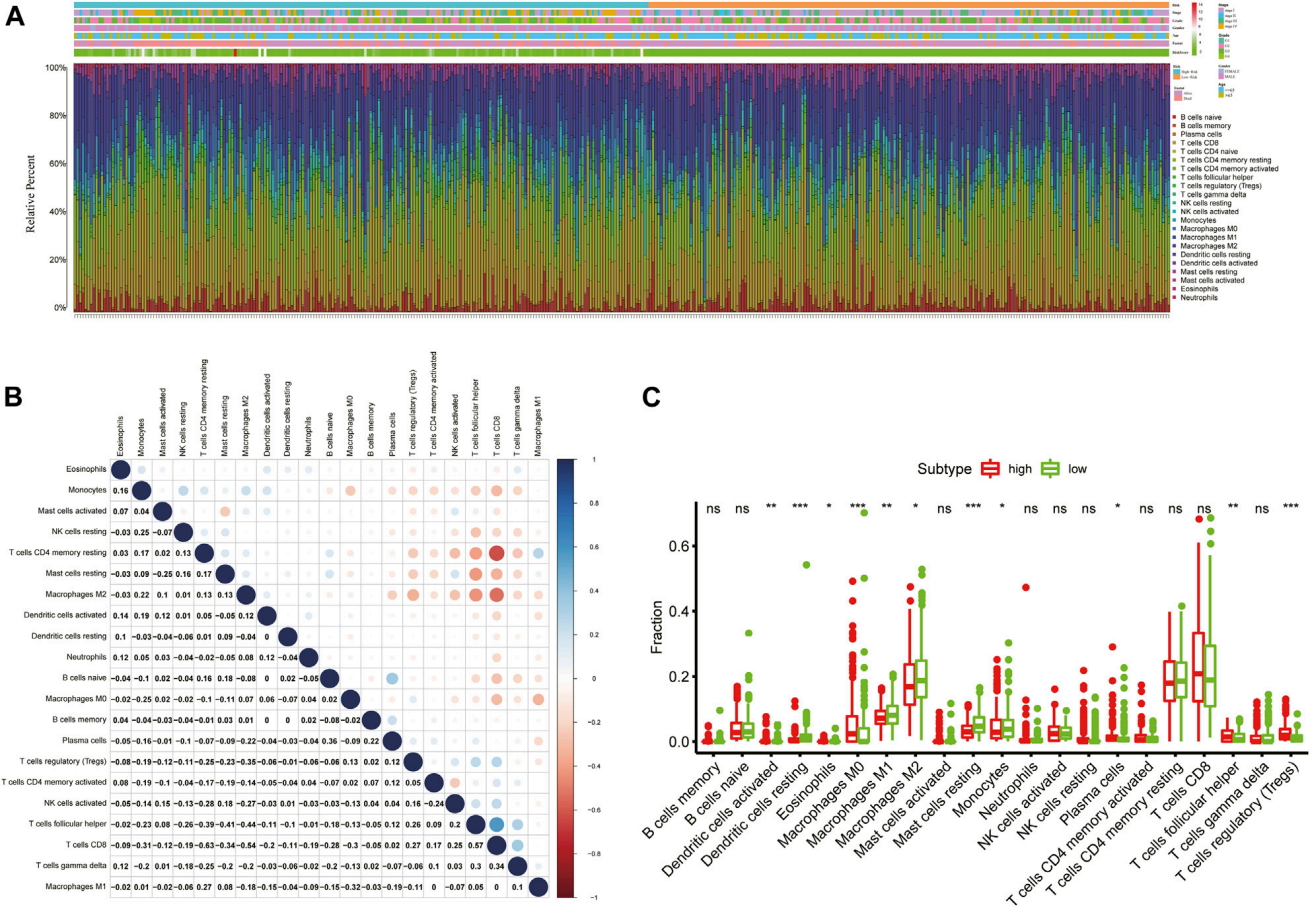
Immune infiltration landscape of KIRC patients in low- and high-risk groups. **(A)** Relative percentage of 22 immune cells in low- and high-risk KIRC patients; **(B)** Correlation analyses between 22 types of immune cells; **(C)** Fractions of 22 immune cells between low- and high-risk groups. *p < 0.05; **p < 0.01; ***p < 0.001.

To obtain comprehensive insights into the infiltration of tumor-infiltrating immune cells in KIRC, the CIBERSORT algorithm combined with the LM22 leukocyte signature matrix was utilized to calculate the proportions of 22 immune cell types in high- and low-risk KIRC patients from the entire cohort. In total, 404 patients from the entire TCGA-KIRC cohort met the inclusion criteria (*p* < 0.05), and their composition of 22 immune cells is summarized in Figure 9A. Besides, the proportions of different tumor-infiltrating immune cell subsets were moderately to weakly correlated (Figure 9B). Between the two groups, the fractions of immune cells in KIRC varied significantly. The KIRC patients in the high-risk group had significantly higher fractions of macrophages M0 (*p* < 0.001), T follicular helper cells (*p* < 0.001), regulatory T cells (*p* < 0.001), dendritic cells activated (*p* < 0.01), and plasma cells (*p* < 0.05), and lower fractions of dendritic cells resting (*p* < 0.001), mast cells resting (*p* < 0.001), macrophages M2 (*p* < 0.01), macrophages M1 (*p* < 0.05), and monocytes (*p* < 0.05) than patients with low risk (Figure 9C).

**FIGURE 10:**
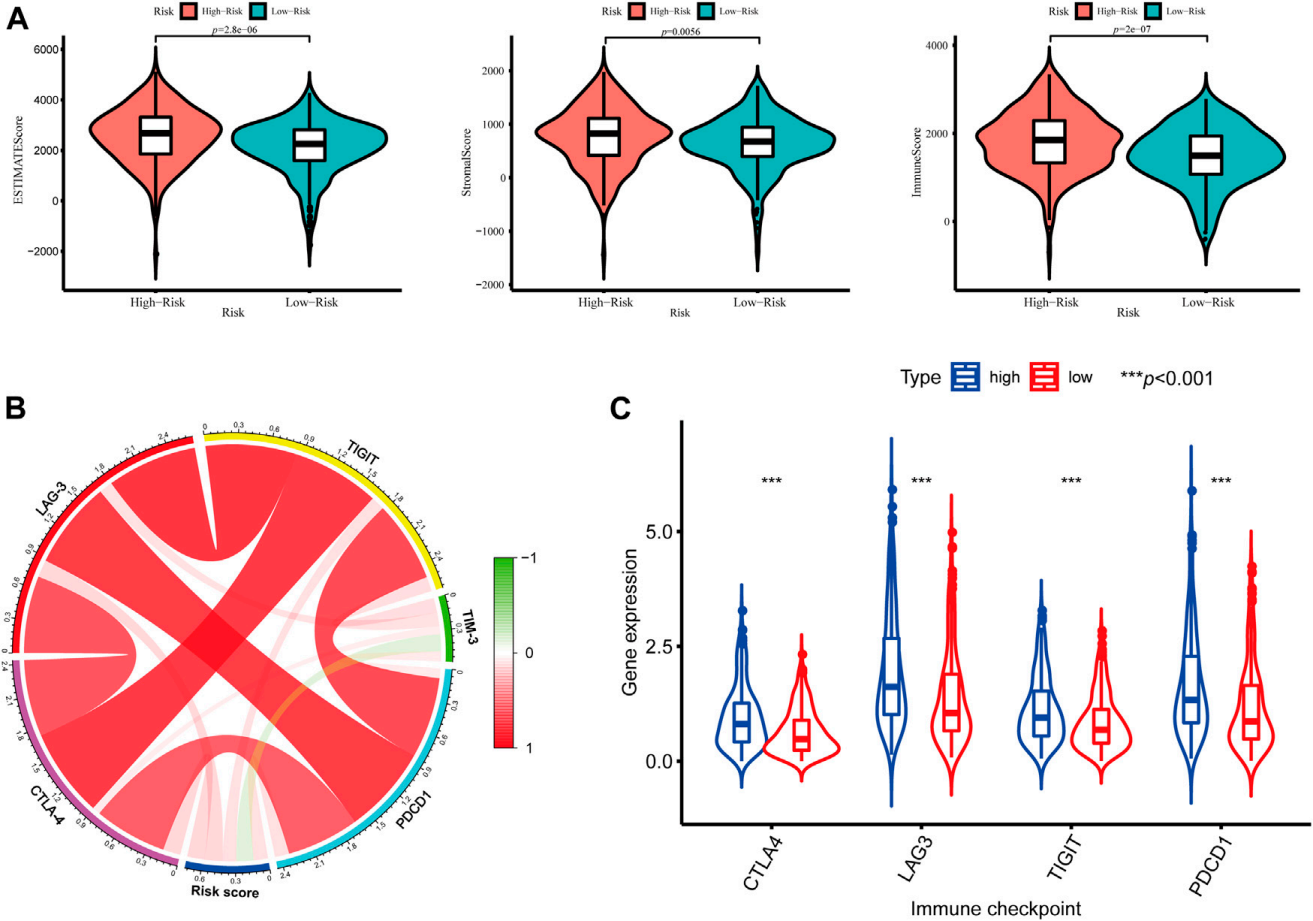
Tumor microenvironment between different risk groups. **(A)** Estimate scores, stromal scores, and immune scores of different groups; **(B)** Correlation between the risk score and the expression of immune checkpoints; **(C)** Significant differentially expressed immune checkpoints between high- and low-risk patients.

In addition, we further assessed the tumor microenvironment of KIRC based on the ESTIMATE method and found that patients in the high-risk group had significantly higher stromal scores (*p* = 0.0056) and immune scores (*p* < 0.001) than those in the low-risk group (Figure 10A). At present, the immune checkpoint blockade therapy has become an important antitumor strategy by reversing the immunosuppressive state. Then, the relationships between the expression levels of several key immune checkpoint molecules (CTLA-4, LAG-3, TIGIT, TIM3, and PDCD1) and risk scores were assessed. We observed that the risk scores were significantly positively associated with the expression of CTLA-4, LAG-3, TIGIT, and PDCD1 (*p* < 0.05) (Figure 10B), and the expression levels of CTLA-4, LAG-3, TIGIT, and PDCD1 were significantly higher in the high-risk KIRC patients than those in the low-risk patients (*p* < 0.001) (Figure 10C).

### A Predictive Nomogram Development and Validation

**FIGURE 11:**
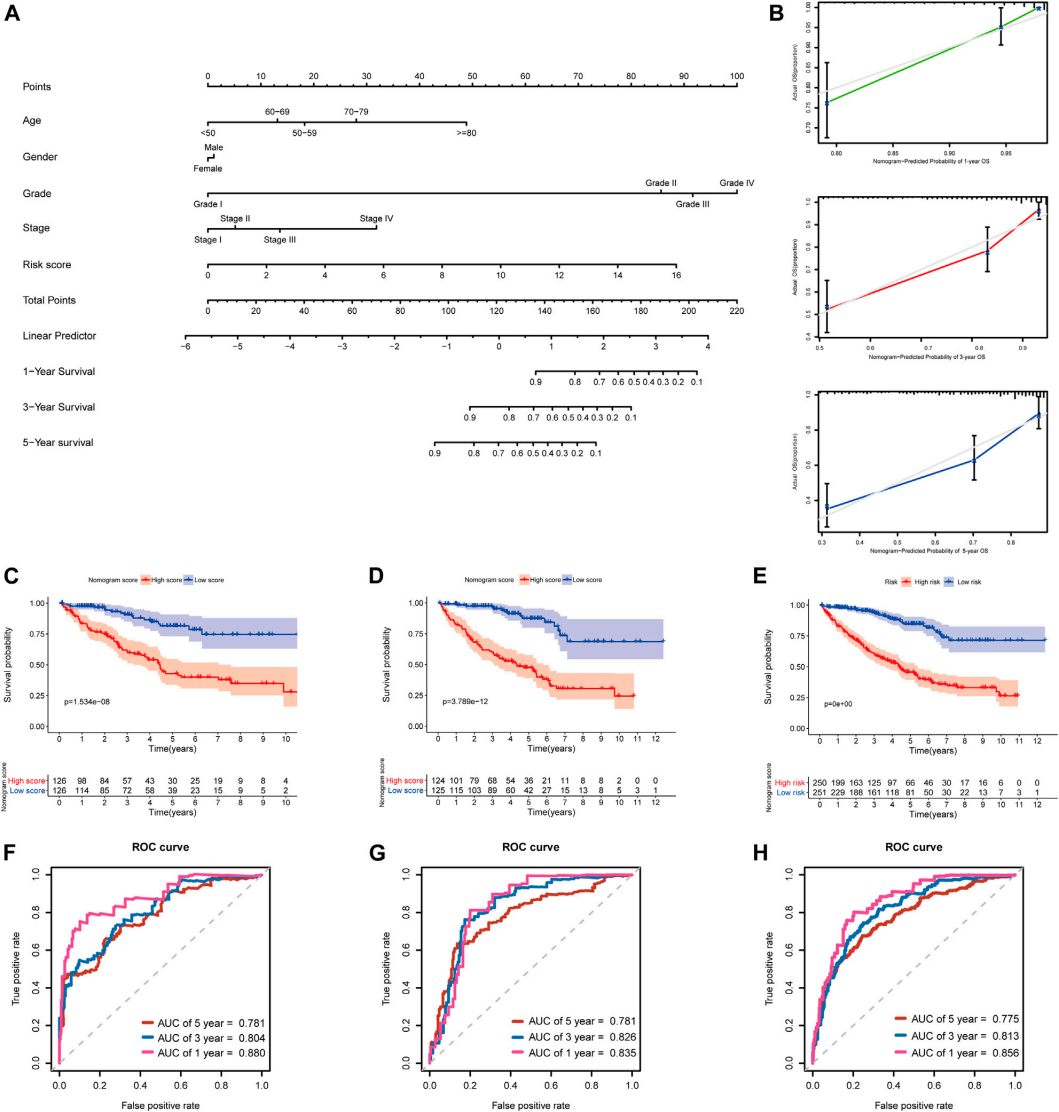
Developing and validating a nomogram for predicting prognosis in the TCGA-KIRC dataset. **(A)** Nomogram constructed in the training set to predict 1-, 3-, and 5-year OS; **(B)** Calibration plots of the nomogram for 1-, 3-, and 5-year OS; **(C–E)** Kaplan–Meier survival analysis based on the nomogram scores in the training set, testing set, and entire TCGA-KIRC cohort; **(F–H)** Time-dependent ROC curves of the nomogram for 1-, 3-, and 5-year OS in the training set, testing set, and entire TCGA-KIRC cohort.

In addition, to further extend the clinical applicability and availability of the prognostic model, a predictive nomogram for 1-, 3-, and 5-year OS combined with age, gender, grade, and risk scores was developed in the training set, where predictors were included in the nomogram (Figure 11A). Calibration plots demonstrated that the nomogram had a similar performance than the ideal model for predicting 1-, 3-, and 5-year OS probability, and there was a good agreement between the predicted and actual observed survival (Figure 11B). The KIRC patients in the training set (*n* = 252) were segregated into high-nomogram score (*n* = 126) and low-nomogram score (*n* = 126) groups determined by the median nomogram score, and Kaplan–Meier analysis indicated that the patients with low-nomogram scores tend to have a significantly worse survival (*p* < 0.001) (Figure 11C). The AUCs of the nomogram in the 1-, 3-, and 5-year ROC curves were 0.880, 0.804, and 0.781, which outperformed the 5-gene signature (Figure 11F; Supplementary Table S5).

Additionally, we further validated the predictive nomogram in the testing set (n = 249) and the entire KIRC cohort (*n* = 501), and similar results were observed in Kaplan–Meier analysis and ROC curve analysis (Figures 11D,E,G–H; Supplementary Table S5). Altogether, these results indicated that the risk score signature in combination with other clinical parameters might have higher predictive power for OS.

### Validation of the 5-Gene Signature and Nomogram in the E-MTAB-1980 Dataset

**FIGURE 12:**
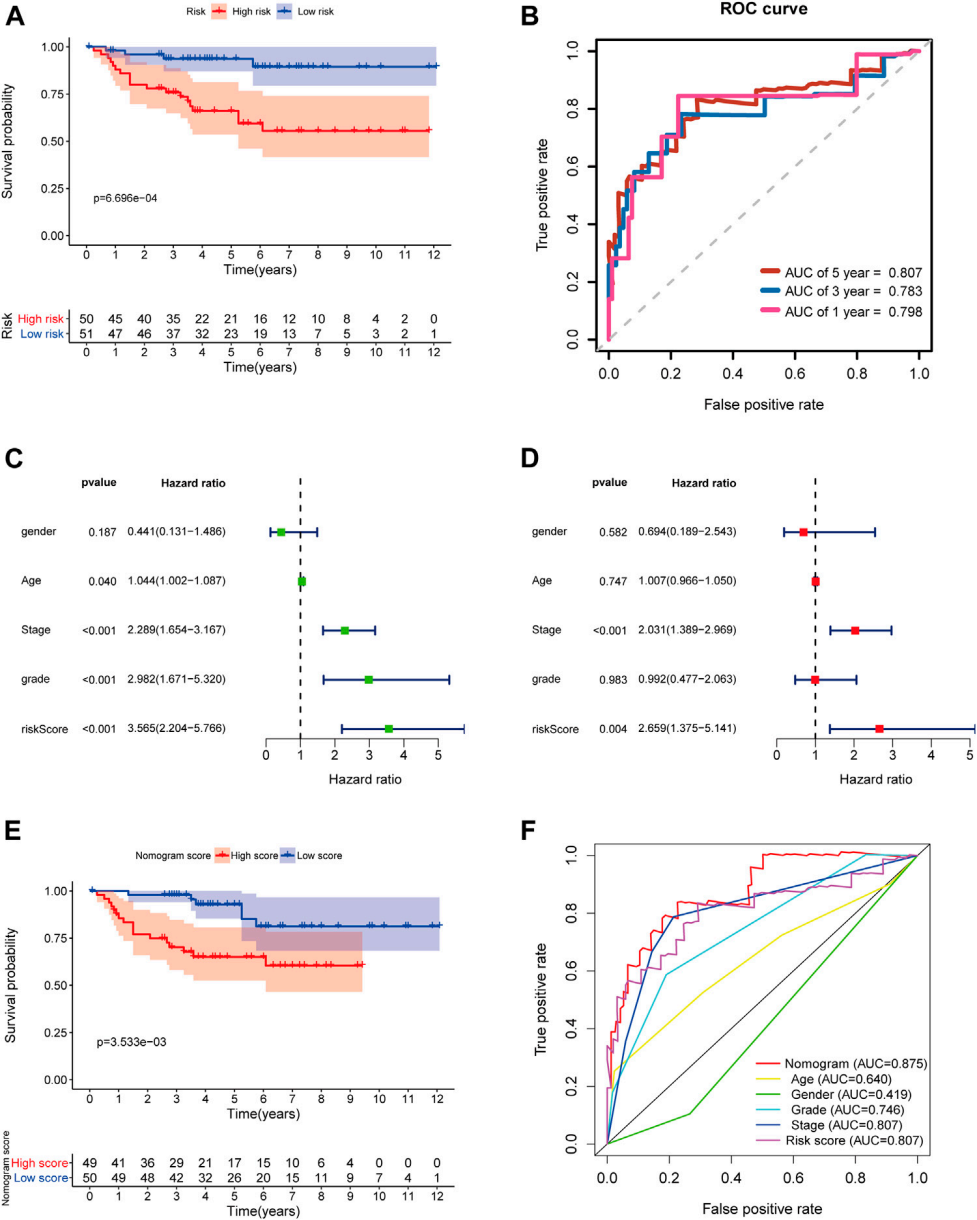
External validation of the prognostic 5-gene signature and nomogram in the E-MTAB-1980 dataset. **(A)** Kaplan–Meier survival analysis based on the 5-gene signature in the E-MTAB-1980 dataset; **(B)** Time-dependent ROC curves of the 5-gene signature for 1-, 3-, and 5-year OS in the E-MTAB-1980 dataset; **(C–D)** Univariate and multivariate Cox regression analyses; **(E)** Kaplan–Meier survival analysis based on the nomogram in the E-MTAB-1980 dataset; **(F)** Time-dependent ROC curves of the nomogram, age, gender, stage, grade, and risk score.

In order to further determine the reliability and robustness of our prognostic signature and nomogram, we used the E-MTAB-1980 dataset for external validation. Table 2 displayed the clinical characteristics of KIRC patients in the E-MTAB-1980 dataset. Risk scores of these patients were calculated using the previous following formula. Similar to our results above, the K-M survival analysis indicated the OS of patients in the high-risk group was significantly shorter than that in the low-risk group (*p* < 0.001) (Figure 12A). The AUC values of the 5-gene prognostic signature for 1-, 3-, and 5-year OS reached 0.798, 0.783, and 0.807, respectively (Figure 12B). Moreover, the univariate and multivariate Cox regression analyses also demonstrated that the 5-gene signature could predict KIRC patients’ prognosis independently (HR = 2.659, 95% CI = 1.375–5.141, *p* = 0.004) in Figures 12C,D. We then validated the nomogram in the E-MTAB-1980 dataset and found that KIRC patients with high nomogram scores had poorer prognosis than those with low nomogram scores (*p* < 0.001) (Figure 12E). The AUC of the nomogram for 5-year OS was 0.875, which exceeded the 5-gene signature and other clinical features (Figure 12F). These results in the E-MTAB-1980 dataset further confirmed the reliability and robustness of the 5-gene prognostic signature and nomogram.

**TABLE 2:** Characteristics of KIRC patients in the external validation cohort (E-MTAB-1980 dataset).

Characteristics		E-MTAB-1980 dataset (*N* = 101)
Status		
	Dead	23 (22.8%)
	Alive	78 (77.2%)
Gender		
	Male	77 (76.2%)
	Female	24 (23.8%)
Age at diagnosis (years)		
	≤65	57 (56.4%)
	>65	44 (43.6%)
Histological grade		
	G1	13 (12.9%)
	G2	59 (58.4%)
	G3	22 (21.8%)
	G4	5 (5.0%)
	Unknown	2 (1.9%)
TNM stage		
	I	66 (65.3%)
	II	10 (9.9%)
	III	13 (12.9%)
	IV	12 (11.9%)

## Discussion

COVID-19 pandemic is becoming a serious global public health problem; the older patients with basic diseases such as hypertension, diabetes, chronic kidney disease, and cancer were considered at an increased risk of COVID-19 infection (Ma et al., 2020; Salzberger et al., 2021). This susceptibility may also be related to the aberrant expression of some key SARS-CoV-2-interacting proteins, especially ACE2 (Li Y. et al., 2020; Ashraf et al., 2021; Rezaei et al., 2021). SARS-CoV-2 utilizes ACE2 as an entry receptor to infect cells, and an increased ACE2 expression may result in an elevated SARS-CoV-2 infection risk (Cao et al., 2020; Wu et al., 2021). Some studies have found that ACE2 is aberrantly expressed in some cancers, such as lung adenocarcinoma, renal carcinoma, and stomach adenocarcinoma, and it is correlated with prognosis and immune infiltrates (Hoang et al., 2020; Kong et al., 2020; Tripathi et al., 2020; Chen L. et al., 2021; He et al., 2021). Moreover, the other key SARS-CoV-2 infection–related host cell receptors or entry related-proteins, TMPRSS2, CTSL/B, USP13, HSPA5, Furin, and ADAM17 have recently been reported to play important roles in SARS-CoV-2 infection (Chen et al., 2020; Ibrahim et al., 2020; Zipeto et al., 2020; Tang et al., 2021). They were also observed to be aberrantly expressed in several cancers; dysregulation of their expression in cancer patients’ tissues should affect the susceptibility and severity of SARS-CoV-2 infection (Li H. et al., 2020; Ravaioli et al., 2020; Süt, 2020; Fu et al., 2021; Zhou and Gao, 2021). These findings suggest that SARS-CoV-2 infection–related genes may play essential roles in virus infection, and they also participate in development of many cancers. To date, over 300 SARS-CoV-2 infection–related genes have been identified, but their roles in KIRC remains unclear. It is important to evaluate their expression in KIRC tissues, for predicting the KIRC patients’ susceptibility to SARS-CoV-2 infection and outcome. Therefore, the goal of our study was to develop a robust SARS-CoV-2–related gene signature, which can predict the clinical outcome for KIRC patients.

In the present study, we systematically analyzed the expression of 333 SARS-CoV-2–related genes in the TCGA-KIRC cohort and found 31 SARS-CoV-2–related DEGs between KIRC and normal tissues. Moreover, we further investigated the association between these SARS-CoV-2–related DEGs and overall survival of KIRC patients by using univariate Cox regression, multiple stepwise Cox regression, and K-M survival analyses and generated a novel prognostic model comprising five SARS-CoV-2–related genes. In the training set, the median risk score was used as the cut-off to categorize KIRC patients into high-risk or low-risk groups, and the high-risk patients had unfavorable prognosis. Multivariate analyses demonstrated that the risk score signature was an independent prognostic factor for OS prediction of KIRC patients. The ROC curves exhibited that the AUC values of the SARS-CoV-2–related gene signature were greater than 0.7 at 1, 3, and 5 years, indicating that the 5-gene signature had a good predictive performance to screen out the KIRC patients with poor prognosis. Additionally, we further validated the risk score model in the testing set, entire TCGA-KIRC cohort, and E-MTAB-1980 dataset and observed the similar results and equally good performance. Stratification analysis suggested that the risk score signature had wide applicability for predicting prognosis of KIRC patients with distinct molecular features and clinicopathological characteristics. The 5-gene signature was able to accurately distinguish the KIRC patients who had a high risk with poor overall survival in different clinical subgroups. All these results indicated that our risk score model was a robust indicator for predicting KIRC patients’ prognosis.

The prognostic signature which we constructed consisted of five SARS-CoV-2–related genes (*ACADM*, *CENPF*, *KDELC1*, *PLOD2*, and *TRMT1*). Of the five genes, *CENPF*, *KDELC1*, *PLOD2*, and *TRMT1* were upregulated in KIRC tissues and were correlated with poor survival and higher susceptibility to SARS-CoV-2. In contrast, *ACADM* was downregulated and act as a protective factor for KIRC patients; the higher *ACADM* expression may contribute to better survival and lower likelihood of SARS-CoV-2 infection. Centromere protein F (*CENPF*), a gene located in chromosome 1q41, encodes a microtubule-binding protein that is associated with the cell cycle and regulate chromosome segregation; it begins to accumulate during the S-phase and reaches maximal expression levels in the G2/M phase cells (Shahid et al., 2018; Sun et al., 2019). *CENPF* has been reported to be upregulated in various types of malignant tumors and serve as a prognostic indicator for multiple cancers, such as prostate cancer, breast cancer, pancreatic cancer, lung adenocarcinoma, and renal cancer; the high expression of *CENPF* is associated with worse prognosis and metastasis in these cancers (Sun et al., 2019; Chen H. et al., 2021). Some studies have found that *CENPF* and *FOXM1* are major regulators in prostate cancer development; they synergistically promote prostate cancer malignant progression and metastasis (Aytes et al., 2014). Moreover, *CENPF* also promotes breast cancer progression and bone metastasis by activating the AKT/mTOR signaling pathway (Sun et al., 2019). Knockdown of *CENPF* could inhibit the progression of lung adenocarcinoma by suppressing the ERβ2/5 pathway (Hexiao et al., 2021). *KDELC1*, also called *POGLUT2*, is a protein O-glucosyltransferase which participates in O-glucose modification and modulates Notch trafficking and signaling (Takeuchi et al., 2018; Mehboob and Lang, 2021). Currently, few research studies are available regarding the role of *KDELC1* in cancer. In this research, we first uncovered that *KDELC1* was upregulated in KIRC and associated with poor survival. *PLOD2* is one of the members of the PLOD family, encodes for lysyl hydroxylases 2which mediates stabilized collagen cross-link formation in the extracellular matrix. Many lines of evidence suggested that *PLOD2* is a tumor metastasis-promoting gene; it promotes tumor aggressive metastasis and invasion through mediating cross-links of collagen (Yamauchi and Sricholpech, 2012; Chen et al., 2015). In recent years, increasing research has revealed the pivotal role of *PLOD2* in various cancer types, particularly in breast cancer, hepatocellular carcinoma, bladder cancer, sarcomas, and renal cell carcinoma (Du et al., 2017; Qi and Xu, 2018). In these cancer tissues, *PLOD2* is significantly overexpressed, and the high *PLOD2* expression was significantly associated with tumor metastasis and shorter survival time. Obviously, *PLOD2* is an independent factor of poor outcomes and could serve as a prognostic biomarker for patients with these cancer types (Du et al., 2017). Several previous studies have shown that the expression *PLOD2* could be regulated by HIF-1α (Gilkes et al., 2013). Besides, in a tumor microenvironment, *PLOD2* could modulate the collagen–cross-linking activity of cancer-associated fibroblasts and promote tumor cell migration and invasion (Chen et al., 2015; Qi and Xu, 2018). *TRMT1* encodes tRNA methyltransferase 1, an RNA methyltransferase responsible for N^2^, N^2^-dimethylguanosine (m2,2G) formation in cytosolic and mitochondrial tRNAs (Dewe et al., 2017; Jonkhout et al., 2021). *TRMT1* has been considered as the cause of autosomal-recessive intellectual disability (Zhang et al., 2020), but its role in cancers has not yet been reported. Herein, our study has reported the prognostic value of *TRMT1* in KIRC for the first time. *ACADM* encodes medium-chain acyl-CoA dehydrogenase which is involved in fatty acid oxidation and lipid metabolism (Smith et al., 2010). Inhibition of *ACADM* could alter hepatocellular carcinoma cell lipid metabolism and promote cell lipid accumulation, ultimately drive hepatocarcinogenesis (Li et al., 2019). Additionally, a recent study showed that *ACADM* was highly expressed in ferret models after SARS-CoV-2 infection and played a crucial role in SARS-CoV-2 infection progression (Liu et al., 2020). In summary, two of the prognostic model genes (*CENPF* and *PLOD2*) have been reported to be associated with KIRC patients’ prognosis in previous studies, while the remaining three genes (*KDELC1*, *TRMT1*, and *ACADM*) were first reported to be correlated with the prognosis of KIRC in the current study.

Evidence has suggested that the immune dysregulation may be highly linked to the pathological process of COVID-19. Given the significance of the immune system in antiviral and antitumor responses, we further investigated the immune cell infiltration status between KIRC patients with low and high risks based on the prognostic signature. The ESTIMATE analysis showed that patients in the high-risk groups were identified to have significantly higher stromal scores and immune scores and lower tumor purities. Recent studies have revealed that major stromal components within the tumor microenvironment can not only favor tumor growth and metastasis but it could also affect the antitumor immune response, leading to unfavorable prognosis (Quail and Joyce, 2013; Turley et al., 2015). Our results were in agreement with these previous findings that high stromal infiltration and low tumor purity levels correlated with worse prognosis in many cancers (Zhang et al., 2017). Furthermore, the CIBERSORT algorithm was used to evaluate the abundance of 22 immune cell types in KIRC, and the higher proportions of macrophages M0, regulatory T cells (Tregs), and T follicular helper cells were observed in high-risk patients than low-risk patients. Tumor-associated macrophages and Treg cells are immunosuppressive cells that inhibit the antitumor immune response and secrete various immunosuppressive cytokines, promoting the tumor immune escape (Dunn et al., 2002; Sica et al., 2006). Moreover, we noted that the high-risk KIRC patients had significantly higher levels of the CTLA-4, LAG-3, TIGIT, and PDCD1 expressions than the low-risk patients, and high expression levels of immune checkpoints are effective predictors for responses to immune checkpoint inhibitors (Pardoll, 2012; Qin et al., 2019). Notably, Treg cells also expressed immune checkpoint molecules, including CTLA-4 and PDCD1 (Romano et al., 2015). The above results indicated that the worse survival of the KIRC patients with high-risk scores is likely due to the higher immune checkpoint expression levels and more potent immunosuppressive tumor microenvironment. Besides, these results also suggested that patients in the high-risk group will benefit more from the immune checkpoint blockade therapy than those in the low-risk group, thereby contributing to a better prognosis.

Additionally, we further established a novel predictive nomogram by integrating our risk signature with several clinical characteristics with superior prediction performance. The AUCs of this nomogram for 1-, 3-, and 5-year survival are all greater than 0.750 in the training set, testing set, entire TCGA-KIRC cohort, and E-MTAB-1980 dataset, which indicated our nomogram and prognostic signature have excellent predictive ability for both short- and long-term follow-up patients. We also found the AUC values of this nomogram exceeded that of the 5-gene signature both in predicting 1-, 3-, and 5-year OS, indicating combination of the 5-gene signature with other prognosis-related clinical parameters would further improve the predictive power for survival and expand the clinical practicability.

To our knowledge, our study for the first time proposed a novel prognostic signature and nomogram for KIRC based upon SARS-CoV-2–related genes and provided some new insights into the relationship between KIRC and SARS-CoV-2 infection. However, some limitations of our study still existed. First, the study was based on retrospective cohorts; our results needed to be further confirmed by future prospective studies. Second, the underlying biological functions and molecular mechanisms of the five model genes in KIRC and SARS-CoV-2 infection have not been examined in this study and remain to be further studied in the future.

In summary, we developed a novel prognostic signature and nomogram based on five SARS-CoV-2–related genes for the first time, which exhibited good performance both in the TCGA cohort and external validation dataset. This prognostic model was confirmed to have independent prognostic significance for KIRC patients and may provide some new potential therapeutic targets for KIRC patients and protects them from SARS-CoV-2 infection.

## Data Availability

The original contributions presented in the study are included in the article/Supplementary Material; further inquiries can be directed to the corresponding author.

## Abbreviations

AUC, area under the curve; CI, confidence interval; COVID-19, coronavirus disease 2019; DEGs, differentially expressed genes; FC, fold change; FDR, false discovery rate; GSEA, gene set enrichment analysis; HPA, human protein atlas; HR, hazard ratio; KIRC, kidney renal clear cell carcinoma; NES, normalized enrichment score; OS, overall survival; PPI, protein–protein interaction; PCA, principal component analysis; RCC, renal cell carcinoma; ROC, receiver operating characteristic; SARS-CoV-2, severe acute respiratory syndrome coronavirus 2; TCGA, The Cancer Genome Atlas.
