# Supplementary material for: A Novel Prognostic Signature for Survival Prediction and Immune Implication Based on SARS-CoV-2–Related Genes in Kidney Renal Clear Cell Carcinoma
Source: Front Bioeng Biotechnol. 2022 Jan 24;9:744659. doi: 10.3389/fbioe.2021.744659 (PMC8819071; doi:10.3389/fbioe.2021.744659)
Supplement: Supplementary file 1 [file DataSheet2.PDF]

Table S1. SARS-CoV2 related genes summarized in HPA database.

Gene name	Covid-19 bait	Tissue specificity	Blood specificity
AAR2	SARS-CoV2 M	Low tissue specificity	Low immune cell specificity
AASS	SARS-CoV2 M	Low tissue specificity	Immune cell enhanced (basophil)
AATF	SARS-CoV2 nsp8	Low tissue specificity	Low immune cell specificity
ABCC1	SARS-CoV2 orf9c	Low tissue specificity	Group enriched (basophil, eosinophil, T-reg)
ACAD9	SARS-CoV2 orf9c	Low tissue specificity	Low immune cell specificity
ACADM	SARS-CoV2 M	Tissue enhanced (liver, skeletal muscle)	Low immune cell specificity
ACE2		Tissue enriched (intestine)	Not detected in immune cells
ACSL3	SARS-CoV2 nsp7	Tissue enriched (parathyroid gland)	Low immune cell specificity
ADAM9	SARS-CoV2 orf8	Low tissue specificity	Immune cell enhanced (classical monocyte)
ADAMTS1	SARS-CoV2 orf8	Tissue enhanced (ovary)	Group enriched (NK-cell, gdT-cell)
AES	SARS-CoV2 nsp13	Low tissue specificity	Low immune cell specificity
AGPS	SARS-CoV2 nsp7	Low tissue specificity	Low immune cell specificity
AKAP8	SARS-CoV2 nsp12	Low tissue specificity	Low immune cell specificity
AKAP8L	SARS-CoV2 M	Low tissue specificity	Low immune cell specificity
AKAP9	SARS-CoV2 nsp13	Low tissue specificity	Low immune cell specificity
ALG11	SARS-CoV2 nsp4	Low tissue specificity	Low immune cell specificity
ALG5	SARS-CoV2 orf3a	Low tissue specificity	Low immune cell specificity
ALG8	SARS-CoV2 orf9c	Low tissue specificity	Low immune cell specificity
ANO6	SARS-CoV2 M	Low tissue specificity	Low immune cell specificity
AP2A2	SARS-CoV2 nsp10	Low tissue specificity	Low immune cell specificity

AP2M1	SARS-CoV2 nsp10	Low tissue specificity	Low immune cell specificity
AP3B1	SARS-CoV2 E	Low tissue specificity	Low immune cell specificity
ARF6	SARS-CoV2 nsp15	Low tissue specificity	Low immune cell specificity
ARL6IP6	SARS-CoV2 orf3a	Tissue enhanced (blood)	Group enriched (basophil, eosinophil)
ATE1	SARS-CoV2 nsp8	Low tissue specificity	Low immune cell specificity
ATP13A3	SARS-CoV2 nsp6	Low tissue specificity	Low immune cell specificity
ATP1B1	SARS-CoV2 M	Tissue enhanced (kidney)	Group enriched (basophil, T-reg, myeloid DC, NK-cell, memory CD4 T-cell, intermediate monocyte, gdT-cell)
ATP5MG	SARS-CoV2 nsp6	Low tissue specificity	Low immune cell specificity
ATP6AP1	SARS-CoV2 nsp6	Low tissue specificity	Low immune cell specificity
ATP6V1A	SARS-CoV2 M	Low tissue specificity	Immune cell enhanced (neutrophil)
BAG5	SARS-CoV2 orf9b	Tissue enhanced (testis)	Low immune cell specificity
BCKDK	SARS-CoV2 nsp12	Low tissue specificity	Low immune cell specificity
BCS1L	SARS-CoV2 orf9c	Low tissue specificity	Low immune cell specificity
BRD2	SARS-CoV2 E	Low tissue specificity	Low immune cell specificity
BRD4	SARS-CoV2 E	Low tissue specificity	Low immune cell specificity
BZW2	SARS-CoV2 M	Tissue enhanced (placenta, skeletal muscle)	Low immune cell specificity
C1orf50	SARS-CoV2 nsp13	Low tissue specificity	Low immune cell specificity
CCDC86	SARS-CoV2 nsp8	Tissue enriched (parathyroid gland)	Low immune cell specificity
CDK5RAP2	SARS-CoV2 nsp13	Low tissue specificity	Low immune cell specificity
CENPF	SARS-CoV2 nsp13	Group enriched (bone marrow, lymphoid tissue)	Low immune cell specificity

CEP112	SARS-CoV2 nsp13	Tissue enhanced (testis)	Low immune cell specificity
CEP135	SARS-CoV2 nsp13	Tissue enhanced (lymphoid tissue)	Low immune cell specificity
CEP250	SARS-CoV2 nsp13	Low tissue specificity	Low immune cell specificity
CEP350	SARS-CoV2 nsp13	Low tissue specificity	Low immune cell specificity
CEP68	SARS-CoV2 nsp13	Low tissue specificity	Low immune cell specificity
CHMP2A	SARS-CoV2 orf9b	Low tissue specificity	Low immune cell specificity
CHPF	SARS-CoV2 orf8	Low tissue specificity	Immune cell enhanced (plasmacytoid DC)
CHPF2	SARS-CoV2 orf8	Low tissue specificity	Low immune cell specificity
CISD3	SARS-CoV2 orf8	Low tissue specificity	Low immune cell specificity
CIT	SARS-CoV2 nsp13	Tissue enhanced (bone marrow, brain)	Immune cell enhanced (T-reg)
CLCC1	SARS-CoV2 orf3a	Low tissue specificity	Low immune cell specificity
CLIP4	SARS-CoV2 nsp13	Low tissue specificity	Low immune cell specificity
CNTRL	SARS-CoV2 nsp13	Low tissue specificity	Low immune cell specificity
COL6A1	SARS-CoV2 orf8	Low tissue specificity	Immune cell enhanced (memory CD8 T-cell, MAIT T-cell)
COLGALT1	SARS-CoV2 nsp1	Low tissue specificity	Low immune cell specificity
COMT	SARS-CoV2 nsp7	Tissue enhanced (liver)	Low immune cell specificity
COQ8B	SARS-CoV2 M	Low tissue specificity	Low immune cell specificity
CRTC3	SARS-CoV2 nsp12	Low tissue specificity	Immune cell enhanced (basophil)
CSDE1	SARS-CoV2 orf9b	Tissue enhanced (skeletal muscle)	Low immune cell specificity
CSNK2A2	SARS-CoV2 N	Low tissue specificity	Low immune cell specificity
CSNK2B	SARS-CoV2 N	Low tissue specificity	Low immune cell specificity

CTSB		Tissue enhanced (thyroid gland)	Group enriched (classical monocyte, intermediate monocyte, plasmacytoid DC, myeloid DC, non-classical monocyte, basophil)
CTSL		Tissue enhanced (placenta)	Group enriched (non-classical monocyte, intermediate monocyte)
CUL2	SARS-CoV2 orf10	Low tissue specificity	Low immune cell specificity
CWC27	SARS-CoV2 E	Low tissue specificity	Low immune cell specificity
CYB5B	SARS-CoV2 nsp7	Tissue enhanced (adrenal gland)	Low immune cell specificity
CYB5R3	SARS-CoV2 nsp7	Low tissue specificity	Low immune cell specificity
DCAF7	SARS-CoV2 nsp9	Low tissue specificity	Low immune cell specificity
DCAKD	SARS-CoV2 nsp7	Low tissue specificity	Low immune cell specificity
DCTPP1	SARS-CoV2 orf9b	Low tissue specificity	Low immune cell specificity
DDX10	SARS-CoV2 nsp8	Low tissue specificity	Low immune cell specificity
DDX21	SARS-CoV2 N	Low tissue specificity	Low immune cell specificity
DNAJC11	SARS-CoV2 nsp4	Low tissue specificity	Low immune cell specificity
DNAJC19	SARS-CoV2 nsp7	Low tissue specificity	Low immune cell specificity
DNMT1	SARS-CoV2 orf8	Low tissue specificity	Low immune cell specificity
DPH5	SARS-CoV2 orf9b	Low tissue specificity	Low immune cell specificity
DPY19L1	SARS-CoV2 orf9c	Low tissue specificity	Low immune cell specificity
ECSIT	SARS-CoV2 orf9c	Low tissue specificity	Low immune cell specificity
EDEM3	SARS-CoV2 orf8	Low tissue specificity	Low immune cell specificity

EIF4E2	SARS-CoV2 nsp2	Low tissue specificity	Low immune cell specificity
EIF4H	SARS-CoV2 nsp9	Low tissue specificity	Low immune cell specificity
ELOB	SARS-CoV2 orf10	Low tissue specificity	Low immune cell specificity
ELOC	SARS-CoV2 orf10	Low tissue specificity	Low immune cell specificity
EMC1	SARS-CoV2 orf8	Low tissue specificity	Low immune cell specificity
ERC1	SARS-CoV2 nsp13	Low tissue specificity	Low immune cell specificity
ERGIC1	SARS-CoV2 nsp10	Low tissue specificity	Low immune cell specificity
ERLEC1	SARS-CoV2 orf8	Low tissue specificity	Low immune cell specificity
ERMP1	SARS-CoV2 orf9c	Tissue enhanced (parathyroid gland)	Immune cell enhanced (NK-cell)
ERO1B	SARS-CoV2 orf8	Tissue enriched (pancreas)	Immune cell enhanced (basophil)
ERP44	SARS-CoV2 orf8	Low tissue specificity	Low immune cell specificity
ETFA	SARS-CoV2 M	Low tissue specificity	Low immune cell specificity
EXOSC2	SARS-CoV2 nsp8	Low tissue specificity	Low immune cell specificity
EXOSC3	SARS-CoV2 nsp8	Low tissue specificity	Low immune cell specificity
EXOSC5	SARS-CoV2 nsp8	Low tissue specificity	Low immune cell specificity
EXOSC8	SARS-CoV2 nsp8	Low tissue specificity	Low immune cell specificity
F2RL1	SARS-CoV2 orf9c	Tissue enhanced (intestine)	Immune cell enriched (neutrophil)
FAM162A	SARS-CoV2 nsp7	Low tissue specificity	Low immune cell specificity
FAM8A1	SARS-CoV2 M	Low tissue specificity	Immune cell enhanced (neutrophil)
FAM98A	SARS-CoV2 N	Low tissue specificity	Low immune cell specificity
FAR2	SARS-CoV2 orf9c	Low tissue specificity	Immune cell enhanced (eosinophil)
FASTKD5	SARS-CoV2 M	Low tissue specificity	Low immune cell specificity

FBLN5	SARS-CoV2 nsp9	Low tissue specificity	Immune cell enhanced (basophil)
FBN1	SARS-CoV2 nsp9	Tissue enhanced (placenta)	Low immune cell specificity
FBN2	SARS-CoV2 nsp9	Tissue enriched (placenta)	Immune cell enhanced (non-classical monocyte, classical monocyte)
FBXL12	SARS-CoV2 orf8	Tissue enhanced (lymphoid tissue)	Low immune cell specificity
FKBP10	SARS-CoV2 orf8	Low tissue specificity	Immune cell enhanced (NK-cell)
FKBP15	SARS-CoV2 nsp2	Low tissue specificity	Low immune cell specificity
FKBP7	SARS-CoV2 orf8	Low tissue specificity	Low immune cell specificity
FOXRED2	SARS-CoV2 orf8	Low tissue specificity	Immune cell enriched (plasmacytoid DC)
FYCO1	SARS-CoV2 nsp13	Tissue enhanced (skeletal muscle)	Low immune cell specificity
G3BP1	SARS-CoV2 N	Low tissue specificity	Low immune cell specificity
G3BP2	SARS-CoV2 N	Low tissue specificity	Low immune cell specificity
GCC1	SARS-CoV2 nsp13	Low tissue specificity	Low immune cell specificity
GCC2	SARS-CoV2 nsp13	Low tissue specificity	Low immune cell specificity
GDF15	SARS-CoV2 orf8	Tissue enriched (placenta)	Immune cell enhanced (neutrophil)
GFER	SARS-CoV2 nsp10	Low tissue specificity	Low immune cell specificity
GGCX	SARS-CoV2 M	Tissue enriched (liver)	Low immune cell specificity
GGH	SARS-CoV2 orf8	Tissue enhanced (kidney, liver)	Immune cell enriched (plasmacytoid DC)
GHITM	SARS-CoV2 orf9c	Low tissue specificity	Low immune cell specificity
GIGYF2	SARS-CoV2 nsp2	Low tissue specificity	Low immune cell specificity
GLA	SARS-CoV2 nsp14	Low tissue specificity	Low immune cell specificity
GNB1	SARS-CoV2 nsp7	Low tissue specificity	Low immune cell specificity

GNG5	SARS-CoV2 nsp7	Low tissue specificity	Low immune cell specificity
GOLGA2	SARS-CoV2 nsp13	Low tissue specificity	Low immune cell specificity
GOLGA3	SARS-CoV2 nsp13	Low tissue specificity	Low immune cell specificity
GOLGA7	SARS-CoV2 Spike	Low tissue specificity	Low immune cell specificity
GOLGB1	SARS-CoV2 nsp13	Low tissue specificity	Low immune cell specificity
GORASP1	SARS-CoV2 nsp13	Low tissue specificity	Low immune cell specificity
GPAA1	SARS-CoV2 orf9c	Low tissue specificity	Low immune cell specificity
GRIPAP1	SARS-CoV2 nsp13	Low tissue specificity	Low immune cell specificity
GRPEL1	SARS-CoV2 nsp10	Low tissue specificity	Low immune cell specificity
GTF2F2	SARS-CoV2 nsp9	Low tissue specificity	Low immune cell specificity
HDAC2	SARS-CoV2 nsp5	Low tissue specificity	Low immune cell specificity
HEATR3	SARS-CoV2 orf7a	Low tissue specificity	Low immune cell specificity
HECTD1	SARS-CoV2 nsp8	Low tissue specificity	Low immune cell specificity
HMOX1	SARS-CoV2 orf3a	Tissue enhanced (lymphoid tissue)	Group enriched (non-classical monocyte, intermediate monocyte, classical monocyte)
HOOK1	SARS-CoV2 nsp13	Tissue enhanced (liver)	Immune cell enhanced (naive CD4 T-cell)
HS2ST1	SARS-CoV2 nsp7	Low tissue specificity	Low immune cell specificity
HS6ST2	SARS-CoV2 orf8	Tissue enhanced (brain, kidney, ovary)	Immune cell enhanced (NK-cell)
HSBP1	SARS-CoV2 nsp13	Low tissue specificity	Low immune cell specificity
HYOU1	SARS-CoV2 orf8	Tissue enhanced (liver)	Immune cell enhanced (plasmacytoid DC)
IDE	SARS-CoV2 nsp4	Low tissue specificity	Low immune cell specificity
IL17RA	SARS-CoV2 orf8	Tissue enhanced	Immune cell enhanced

		(lymphoid tissue)	(neutrophil)
IMPDH2	SARS-CoV2 nsp14	Low tissue specificity	Low immune cell specificity
INHBE	SARS-CoV2 orf8	Tissue enriched (liver)	Immune cell enhanced (neutrophil)
INTS4	SARS-CoV2 M	Low tissue specificity	Low immune cell specificity
ITGB1	SARS-CoV2 orf8	Low tissue specificity	Low immune cell specificity
JAKMIP1	SARS-CoV2 nsp13	Tissue enhanced (blood, brain)	Group enriched (T-reg, gdT-cell, memory CD8 T-cell, memory CD4 T-cell, MAIT T-cell, naive CD8 T-cell)
KDELC1	SARS-CoV2 orf8	Low tissue specificity	Immune cell enhanced (NK-cell)
KDELC2	SARS-CoV2 orf8	Tissue enhanced (placenta)	Low immune cell specificity
LARP1	SARS-CoV2 N	Low tissue specificity	Low immune cell specificity
LARP4B	SARS-CoV2 nsp12	Low tissue specificity	Low immune cell specificity
LARP7	SARS-CoV2 nsp8	Low tissue specificity	Low immune cell specificity
LMAN2	SARS-CoV2 nsp7	Low tissue specificity	Low immune cell specificity
LOX	SARS-CoV2 orf8	Low tissue specificity	Not detected in immune cells
MAP7D1	SARS-CoV2 orf10	Tissue enhanced (skeletal muscle)	Immune cell enhanced (neutrophil)
1-Mar	SARS-CoV2 nsp7	Tissue enhanced (adipose tissue, breast)	Group enriched (neutrophil, classical monocyte, basophil, eosinophil)
MARK1	SARS-CoV2 orf9b	Low tissue specificity	Not detected in immune cells
MARK2	SARS-CoV2 orf9b	Low tissue specificity	Low immune cell specificity
MARK3	SARS-CoV2 orf9b	Low tissue specificity	Immune cell enriched (eosinophil)
MAT2B	SARS-CoV2 nsp9	Low tissue specificity	Low immune cell specificity
MDN1	SARS-CoV2 orf7a	Low tissue specificity	Low immune cell specificity

MEPCE	SARS-CoV2 nsp8	Low tissue specificity	Low immune cell specificity
MFGE8	SARS-CoV2 orf8	Low tissue specificity	Immune cell enhanced (T-reg)
MIB1	SARS-CoV2 nsp9	Low tissue specificity	Low immune cell specificity
MIPOL1	SARS-CoV2 nsp13	Low tissue specificity	Immune cell enhanced (neutrophil)
MOGS	SARS-CoV2 nsp7	Low tissue specificity	Low immune cell specificity
MOV10	SARS-CoV2 N	Low tissue specificity	Low immune cell specificity
MPHOSPH10	SARS-CoV2 nsp8	Low tissue specificity	Low immune cell specificity
MRPS2	SARS-CoV2 nsp8	Low tissue specificity	Low immune cell specificity
MRPS25	SARS-CoV2 nsp8	Low tissue specificity	Low immune cell specificity
MRPS27	SARS-CoV2 nsp8	Low tissue specificity	Low immune cell specificity
MRPS5	SARS-CoV2 nsp8	Low tissue specificity	Low immune cell specificity
MTCH1	SARS-CoV2 orf6	Low tissue specificity	Low immune cell specificity
MYCBP2	SARS-CoV2 nsp12	Low tissue specificity	Low immune cell specificity
NARS2	SARS-CoV2 nsp8	Low tissue specificity	Low immune cell specificity
NAT14	SARS-CoV2 nsp7	Tissue enhanced (lymphoid tissue)	Low immune cell specificity
NDFIP2	SARS-CoV2 orf9c	Low tissue specificity	Immune cell enhanced (eosinophil)
NDUFAF1	SARS-CoV2 orf9c	Low tissue specificity	Low immune cell specificity
NDUFAF2	SARS-CoV2 nsp7	Low tissue specificity	Low immune cell specificity
NDUFB9	SARS-CoV2 orf9c	Tissue enhanced (skeletal muscle)	Low immune cell specificity
NEK9	SARS-CoV2 nsp9	Low tissue specificity	Low immune cell specificity
NEU1	SARS-CoV2 orf8	Low tissue specificity	Low immune cell specificity
NGDN	SARS-CoV2 nsp8	Low tissue specificity	Low immune cell specificity

NGLY1	SARS-CoV2 orf8	Low tissue specificity	Low immune cell specificity
NIN	SARS-CoV2 nsp13	Low tissue specificity	Low immune cell specificity
NINL	SARS-CoV2 nsp13	Low tissue specificity	Immune cell enhanced (naive CD8 T-cell)
NLRX1	SARS-CoV2 orf9c	Tissue enhanced (esophagus, lymphoid tissue, tongue)	Immune cell enhanced (eosinophil)
NOL10	SARS-CoV2 nsp8	Low tissue specificity	Low immune cell specificity
NPC2	SARS-CoV2 orf8	Low tissue specificity	Low immune cell specificity
NPTX1	SARS-CoV2 orf8	Tissue enriched (brain)	Immune cell enriched (NK-cell)
NSD2	SARS-CoV2 nsp8	Low tissue specificity	Low immune cell specificity
NUP214	SARS-CoV2 nsp9	Tissue enhanced (blood)	Group enriched (neutrophil, classical monocyte)
NUP54	SARS-CoV2 nsp9	Low tissue specificity	Low immune cell specificity
NUP58	SARS-CoV2 nsp9	Low tissue specificity	Low immune cell specificity
NUP62	SARS-CoV2 nsp9	Low tissue specificity	Low immune cell specificity
NUP88	SARS-CoV2 nsp9	Low tissue specificity	Low immune cell specificity
NUP98	SARS-CoV2 orf6	Low tissue specificity	Low immune cell specificity
NUTF2	SARS-CoV2 nsp15	Low tissue specificity	Low immune cell specificity
OS9	SARS-CoV2 orf8	Low tissue specificity	Low immune cell specificity
PABPC1	SARS-CoV2 N	Low tissue specificity	Low immune cell specificity
PABPC4	SARS-CoV2 N	Tissue enhanced (pancreas, skeletal muscle)	Low immune cell specificity
PCNT	SARS-CoV2 nsp13	Tissue enriched (skeletal muscle)	Immune cell enhanced (naive CD8 T-cell)
PCSK6	SARS-CoV2 orf8	Tissue enhanced (brain, liver, lymphoid tissue)	Immune cell enhanced (NK-cell)
PDE4DIP	SARS-CoV2 nsp13	Group enriched (heart muscle, skeletal muscle)	Low immune cell specificity

PDZD11	SARS-CoV2 nsp12	Low tissue specificity	Low immune cell specificity
PIGO	SARS-CoV2 orf9c	Low tissue specificity	Low immune cell specificity
PIGS	SARS-CoV2 orf9c	Low tissue specificity	Low immune cell specificity
PITRM1	SARS-CoV2 M	Low tissue specificity	Low immune cell specificity
PKP2	SARS-CoV2 nsp1	Tissue enriched (heart muscle)	Immune cell enhanced (eosinophil)
PLAT	SARS-CoV2 orf8	Tissue enhanced (parathyroid gland)	Immune cell enhanced (T-reg, memory CD4 T-cell)
PLD3	SARS-CoV2 orf8	Low tissue specificity	Immune cell enriched (basophil)
PLEKHA5	SARS-CoV2 nsp12	Low tissue specificity	Immune cell enhanced (basophil)
PLEKHF2	SARS-CoV2 orf8	Low tissue specificity	Low immune cell specificity
PLOD2	SARS-CoV2 orf8	Tissue enhanced (liver)	Immune cell enhanced (neutrophil)
PMPCA	SARS-CoV2 M	Low tissue specificity	Low immune cell specificity
PMPCB	SARS-CoV2 M	Low tissue specificity	Low immune cell specificity
POFUT1	SARS-CoV2 orf8	Low tissue specificity	Low immune cell specificity
POLA1	SARS-CoV2 nsp1	Low tissue specificity	Low immune cell specificity
POLA2	SARS-CoV2 nsp1	Low tissue specificity	Low immune cell specificity
POR	SARS-CoV2 nsp2	Tissue enhanced (liver)	Low immune cell specificity
PPIL3	SARS-CoV2 nsp12	Low tissue specificity	Low immune cell specificity
PPT1	SARS-CoV2 orf10	Low tissue specificity	Low immune cell specificity
PRIM1	SARS-CoV2 nsp1	Low tissue specificity	Low immune cell specificity
PRIM2	SARS-CoV2 nsp1	Tissue enhanced (lymphoid tissue, prostate)	Low immune cell specificity
PRKACA	SARS-CoV2 nsp13	Low tissue specificity	Low immune cell specificity
PRKAR2A	SARS-CoV2 nsp13	Tissue enhanced (testis)	Low immune cell

			specificity
PRKAR2B	SARS-CoV2 nsp13	Group enriched (adipose tissue, breast)	Immune cell enhanced (myeloid DC)
PRRC2B	SARS-CoV2 nsp12	Low tissue specificity	Low immune cell specificity
PSMD8	SARS-CoV2 M	Low tissue specificity	Low immune cell specificity
PTBP2	SARS-CoV2 orf9b	Low tissue specificity	Low immune cell specificity
PTGES2	SARS-CoV2 nsp7	Tissue enhanced (skeletal muscle)	Low immune cell specificity
PUSL1	SARS-CoV2 orf8	Low tissue specificity	Low immune cell specificity
PVR	SARS-CoV2 orf8	Tissue enhanced (liver)	Group enriched (non-classical monocyte, intermediate monocyte, classical monocyte, myeloid DC)
QSOX2	SARS-CoV2 nsp7	Low tissue specificity	Immune cell enhanced (naive B-cell)
RAB10	SARS-CoV2 nsp7	Low tissue specificity	Low immune cell specificity
RAB14	SARS-CoV2 nsp7	Low tissue specificity	Low immune cell specificity
RAB18	SARS-CoV2 nsp7	Low tissue specificity	Low immune cell specificity
RAB1A	SARS-CoV2 nsp7	Low tissue specificity	Low immune cell specificity
RAB2A	SARS-CoV2 nsp7	Low tissue specificity	Low immune cell specificity
RAB5C	SARS-CoV2 nsp7	Low tissue specificity	Low immune cell specificity
RAB7A	SARS-CoV2 nsp7	Low tissue specificity	Low immune cell specificity
RAB8A	SARS-CoV2 nsp7	Low tissue specificity	Low immune cell specificity
RAE1	SARS-CoV2 orf6	Tissue enhanced (testis)	Low immune cell specificity
RALA	SARS-CoV2 nsp7	Low tissue specificity	Low immune cell specificity
RAP1GDS1	SARS-CoV2 nsp2	Low tissue specificity	Low immune cell specificity

RBM28	SARS-CoV2 N	Low tissue specificity	Low immune cell specificity
RBM41	SARS-CoV2 nsp12	Low tissue specificity	Low immune cell specificity
RBX1	SARS-CoV2 orf10	Low tissue specificity	Low immune cell specificity
RDX	SARS-CoV2 nsp13	Tissue enhanced (adrenal gland)	Low immune cell specificity
REEP5	SARS-CoV2 M	Low tissue specificity	Low immune cell specificity
REEP6	SARS-CoV2 M	Tissue enhanced (intestine, liver, testis)	Low immune cell specificity
RETREG3	SARS-CoV2 orf9c	Low tissue specificity	Low immune cell specificity
RHOA	SARS-CoV2 nsp7	Low tissue specificity	Low immune cell specificity
RIPK1	SARS-CoV2 nsp12	Low tissue specificity	Low immune cell specificity
RNF41	SARS-CoV2 nsp15	Low tissue specificity	Low immune cell specificity
RPL36	SARS-CoV2 N	Low tissue specificity	Low immune cell specificity
RRP9	SARS-CoV2 N	Low tissue specificity	Low immune cell specificity
RTN4	SARS-CoV2 M	Low tissue specificity	Low immune cell specificity
SAAL1	SARS-CoV2 M	Low tissue specificity	Low immune cell specificity
SBNO1	SARS-CoV2 nsp12	Tissue enhanced (testis)	Low immune cell specificity
SCAP	SARS-CoV2 orf9c	Low tissue specificity	Low immune cell specificity
SCARB1	SARS-CoV2 nsp7	Tissue enhanced (adrenal gland, liver, placenta)	Immune cell enhanced (plasmacytoid DC)
SCCPDH	SARS-CoV2 nsp7	Tissue enhanced (blood)	Immune cell enriched (basophil)
SDF2	SARS-CoV2 orf8	Low tissue specificity	Low immune cell specificity
SELENOS	SARS-CoV2 nsp7	Low tissue specificity	Immune cell enriched (plasmacytoid DC)
SEPSECS	SARS-CoV2 nsp8	Tissue enhanced (liver)	Low immune cell specificity
SIGMAR1	SARS-CoV2 nsp6	Tissue enhanced (liver)	Low immune cell specificity

SIL1	SARS-CoV2 orf8	Low tissue specificity	Low immune cell specificity
SIRT5	SARS-CoV2 nsp14	Low tissue specificity	Low immune cell specificity
SLC25A21	SARS-CoV2 M	Tissue enhanced (testis)	Not detected in immune cells
SLC27A2	SARS-CoV2 nsp2	Tissue enriched (liver)	Immune cell enriched (basophil)
SLC30A6	SARS-CoV2 orf9c	Low tissue specificity	Low immune cell specificity
SLC30A7	SARS-CoV2 M	Low tissue specificity	Low immune cell specificity
SLC30A9	SARS-CoV2 M	Low tissue specificity	Low immune cell specificity
SLC44A2	SARS-CoV2 E	Low tissue specificity	Low immune cell specificity
SLC9A3R1	SARS-CoV2 orf9b	Low tissue specificity	Low immune cell specificity
SLU7	SARS-CoV2 nsp12	Low tissue specificity	Low immune cell specificity
SMOC1	SARS-CoV2 orf8	Tissue enhanced (brain, liver)	Immune cell enriched (plasmacytoid DC)
SNIP1	SARS-CoV2 N	Low tissue specificity	Low immune cell specificity
SPART	SARS-CoV2 nsp9	Low tissue specificity	Immune cell enriched (basophil)
SRP19	SARS-CoV2 nsp8	Low tissue specificity	Low immune cell specificity
SRP54	SARS-CoV2 nsp8	Low tissue specificity	Low immune cell specificity
SRP72	SARS-CoV2 nsp8	Low tissue specificity	Low immune cell specificity
STC2	SARS-CoV2 orf8	Tissue enhanced (breast, pancreas)	Not detected in immune cells
STOM	SARS-CoV2 M	Low tissue specificity	Low immune cell specificity
STOML2	SARS-CoV2 orf3b	Low tissue specificity	Low immune cell specificity
SUN2	SARS-CoV2 orf3a	Low tissue specificity	Low immune cell specificity
TAPT1	SARS-CoV2 orf9c	Low tissue specificity	Immune cell enhanced (naive B-cell)
TARS2	SARS-CoV2 M	Low tissue specificity	Low immune cell specificity

TBCA	SARS-CoV2 nsp11	Low tissue specificity	Low immune cell specificity
TBK1	SARS-CoV2 nsp13	Low tissue specificity	Low immune cell specificity
TBKBP1	SARS-CoV2 nsp13	Low tissue specificity	Immune cell enhanced (neutrophil)
TCF12	SARS-CoV2 nsp12	Tissue enhanced (lymphoid tissue)	Low immune cell specificity
THTPA	SARS-CoV2 orf10	Low tissue specificity	Low immune cell specificity
TIMM10	SARS-CoV2 nsp4	Low tissue specificity	Low immune cell specificity
TIMM10B	SARS-CoV2 nsp4	Low tissue specificity	Low immune cell specificity
TIMM29	SARS-CoV2 nsp4	Low tissue specificity	Low immune cell specificity
TIMM8B	SARS-CoV2 orf10	Low tissue specificity	Low immune cell specificity
TIMM9	SARS-CoV2 nsp4	Low tissue specificity	Low immune cell specificity
TLE1	SARS-CoV2 nsp13	Low tissue specificity	Low immune cell specificity
TLE3	SARS-CoV2 nsp13	Low tissue specificity	Immune cell enriched (neutrophil)
TM2D3	SARS-CoV2 orf8	Low tissue specificity	Low immune cell specificity
TMED5	SARS-CoV2 orf9c	Low tissue specificity	Low immune cell specificity
TMEM39B	SARS-CoV2 orf9c	Low tissue specificity	Low immune cell specificity
TMEM97	SARS-CoV2 orf9c	Tissue enriched (pancreas)	Low immune cell specificity
TMPRSS2		Tissue enhanced (intestine, pancreas, prostate)	Not detected in immune cells
TOMM70	SARS-CoV2 orf9b	Low tissue specificity	Low immune cell specificity
TOR1A	SARS-CoV2 orf8	Low tissue specificity	Low immune cell specificity
TOR1AIP1	SARS-CoV2 nsp7	Low tissue specificity	Low immune cell specificity
TRIM59	SARS-CoV2 orf3a	Tissue enhanced (brain, lymphoid tissue)	Low immune cell specificity
TRMT1	SARS-CoV2 nsp5_C145A	Low tissue specificity	Low immune cell specificity

TUBGCP2	SARS-CoV2 M	Low tissue specificity	Low immune cell specificity
TUBGCP3	SARS-CoV2 M	Low tissue specificity	Low immune cell specificity
TYSND1	SARS-CoV2 nsp12	Tissue enhanced (testis)	Low immune cell specificity
UBAP2	SARS-CoV2 nsp12	Low tissue specificity	Low immune cell specificity
UBAP2L	SARS-CoV2 nsp12	Low tissue specificity	Low immune cell specificity
UBXN8	SARS-CoV2 orf9c	Tissue enhanced (ovary)	Low immune cell specificity
UGGT2	SARS-CoV2 orf8	Low tissue specificity	Low immune cell specificity
UPF1	SARS-CoV2 N	Low tissue specificity	Low immune cell specificity
USP13	SARS-CoV2 nsp13	Tissue enhanced (heart muscle, skeletal muscle)	Immune cell enriched (MAIT T-cell)
USP54	SARS-CoV2 nsp12	Tissue enhanced (brain)	Low immune cell specificity
VPS11	SARS-CoV2 orf3a	Low tissue specificity	Low immune cell specificity
VPS39	SARS-CoV2 orf3a	Low tissue specificity	Low immune cell specificity
WASHC4	SARS-CoV2 nsp2	Low tissue specificity	Low immune cell specificity
WFS1	SARS-CoV2 orf9c	Low tissue specificity	Low immune cell specificity
YIF1A	SARS-CoV2 M	Tissue enhanced (liver)	Low immune cell specificity
ZC3H18	SARS-CoV2 E	Low tissue specificity	Low immune cell specificity
ZC3H7A	SARS-CoV2 nsp12	Low tissue specificity	Low immune cell specificity
ZDHHC5	SARS-CoV2 Spike	Low tissue specificity	Low immune cell specificity
ZNF318	SARS-CoV2 nsp12	Low tissue specificity	Immune cell enriched (naive B-cell)
ZNF503	SARS-CoV2 nsp9	Low tissue specificity	Immune cell enhanced (classical monocyte, myeloid DC)
ZYG11B	SARS-CoV2 orf10	Tissue enhanced (skeletal muscle)	Low immune cell specificity

Table S2. The coefficients of the five SARS-CoV-2 interacting protein genes.

Gene symbol	Coefficient	HR	Lower 95% CI	Upper 95% CI	P-value
ACADM	-0.4955	0.6092	0.4337	0.8558	0.0043
CENPF	0.3805	1.4630	0.9789	2.1864	0.0634
KDELC1	0.2904	1.3370	0.9591	1.8639	0.0866
PLOD2	0.2048	1.2272	0.9606	1.5679	0.1014
TRMT1	0.4925	1.6364	1.0220	2.6203	0.0403

HR: hazard ratio, CI: confidence interval.

Table S3. The five prognostic SARS-CoV-2 interacting proteins identified by Cox regression analysis in training set.

Gene name	Covid-19 bait	Tissue specificity	Blood specificity	Subcellular location (main)
ACADM	SARS-CoV2 M	Tissue enhanced (liver, skeletal muscle)	Low immune cell specificity	Mitochondria
CENPF	SARS-CoV2 nsp13	Group enriched (bone marrow, lymphoid tissue)	Low immune cell specificity	Nucleoplasm
KDELC1	SARS-CoV2 orf8	Low tissue specificity	Immune cell enhanced (NK-cell)	Nucleoplasm
PLOT2	SARS-CoV2 orf8	Tissue enhanced (liver)	Immune cell enhanced (neutrophil)	Nucleoli, Cytosol
TRMT1	SARS-CoV2 nsp5_C145A	Low tissue specificity	Low immune cell specificity	Plasma membrane, Cytosol and additionally in Nucleoplasm

Table S4. Univariate and multivariate regression analyses in training, testing, and all TCGA KIRC cohorts.

Variables	Univariate Cox analysis			Multivariate Cox regression		
	**HR**	**95%CI**	**P-value**	**HR**	**95%CI**	**P-value**
**Training cohort**						
Age	1.032	1.012-1.051	**0.001**	1.040	1.017-1.063	**<0.001**
Gender	1.171	0.746-1.838	0.492	1.028	0.636-1.660	0.911
Histological grade	2.252	1.650-3.074	**<0.001**	1.378	0.987-1.924	0.060
TNM stage	1.787	1.477-2.163	**<0.001**	1.556	1.253-1.932	**<0.001**
5-gene Risk score	1.250	1.171-1.334	**<0.001**	1.302	1.196-1.417	**<0.001**
**Testing cohort**						
Age	1.026	1.008-1.045	**0.006**	1.033	1.012-1.055	**0.002**
Gender	0.923	0.590-1.442	0.725	0.956	0.602-1.518	0.849
Histological grade	2.303	1.749-3.032	**<0.001**	1.365	0.990-1.882	0.058
TNM stage	1.990	1.650-2.402	**<0.001**	1.790	1.439-2.228	**<0.001**
5-gene Risk score	1.126	1.074-1.180	**<0.001**	1.142	1.072-1.218	**<0.001**
**Entire TCGA KIRC cohort**						
Age	1.029	1.015-1.042	**<0.001**	1.035	1.019-1.050	**<0.001**
Gender	1.039	0.757-1.426	0.813	0.953	0.686-1.324	0.774
Histological grade	2.267	1.844-2.786	**<0.001**	1.346	1.068-1.697	**0.012**
TNM stage	1.895	1.658-2.168	**<0.001**	1.726	1.483-2.010	**<0.001**
5-gene Risk score	1.151	1.111-1.193	**<0.001**	1.175	1.122-1.231	**<0.001**

HR: hazard ratio, CI: confidence interval.

Table S5. Area under curve (AUC) values from time-dependent receiver operating characteristic (ROC) curves.

	AUC	
	**5-gene Risk signature**	**Nomogram**
**Training set**		
1-Year	0.766	0.880
3-Year	0.705	0.804
5-Year	0.770	0.781
**Testing set**		
1-Year	0.741	0.835
3-Year	0.715	0.826
5-Year	0.712	0.781
**Entire TCGA-KIRC cohort**		
1-Year	0.752	0.856
3-Year	0.707	0.813
5-Year	0.735	0.775
