# Supplementary material for: A Novel Prognostic Signature for Survival Prediction and Immune Implication Based on SARS-CoV-2–Related Genes in Kidney Renal Clear Cell Carcinoma
Source: Front Bioeng Biotechnol. 2022 Jan 24;9:744659. doi: 10.3389/fbioe.2021.744659 (PMC8819071; doi:10.3389/fbioe.2021.744659)
Supplement: Supplementary file 2 [file DataSheet1.PDF]

Supplementary figures

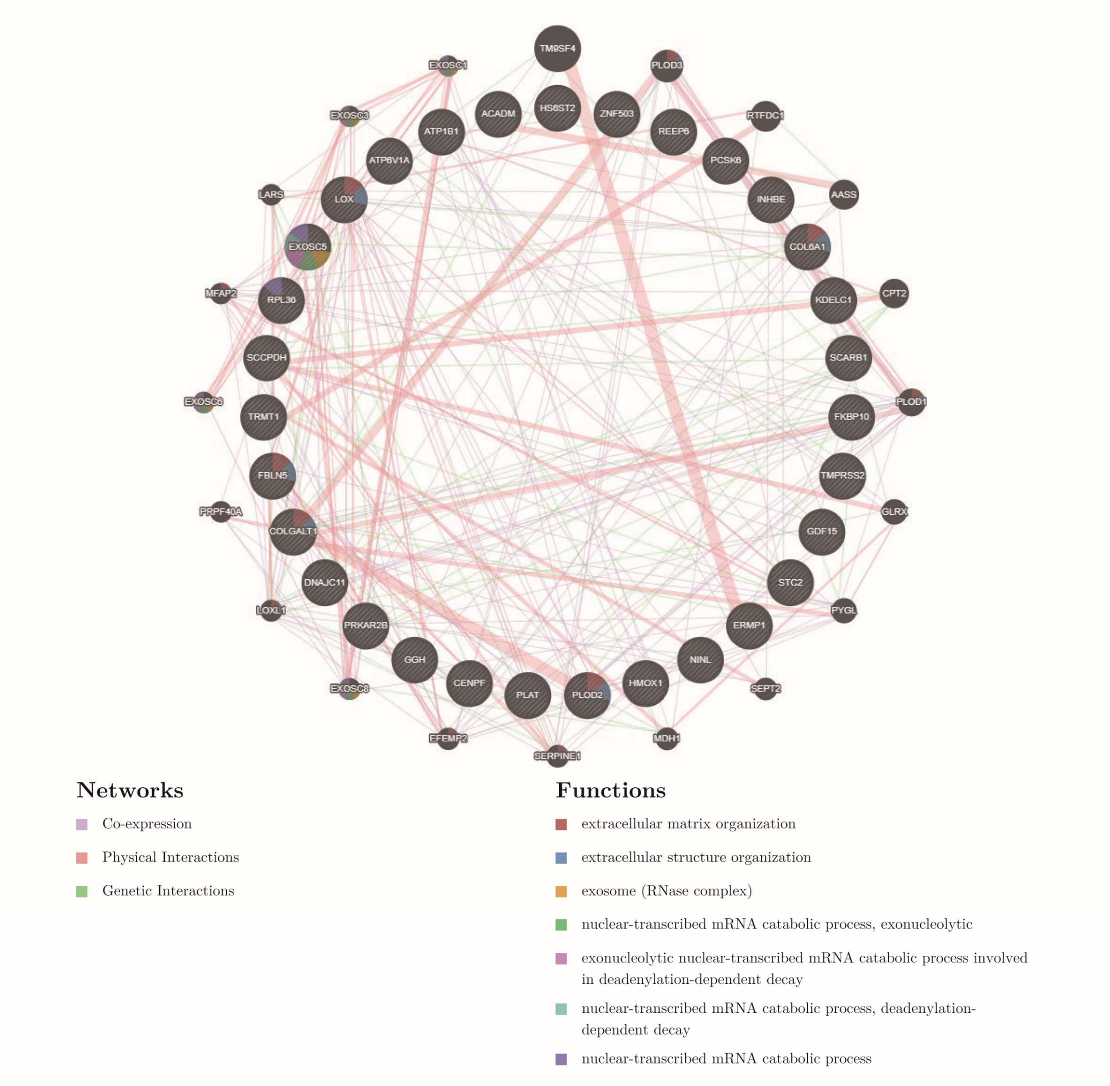

Figure S1. The interaction network of SARS-CoV-2 related DEGs generated by GeneMANIA.

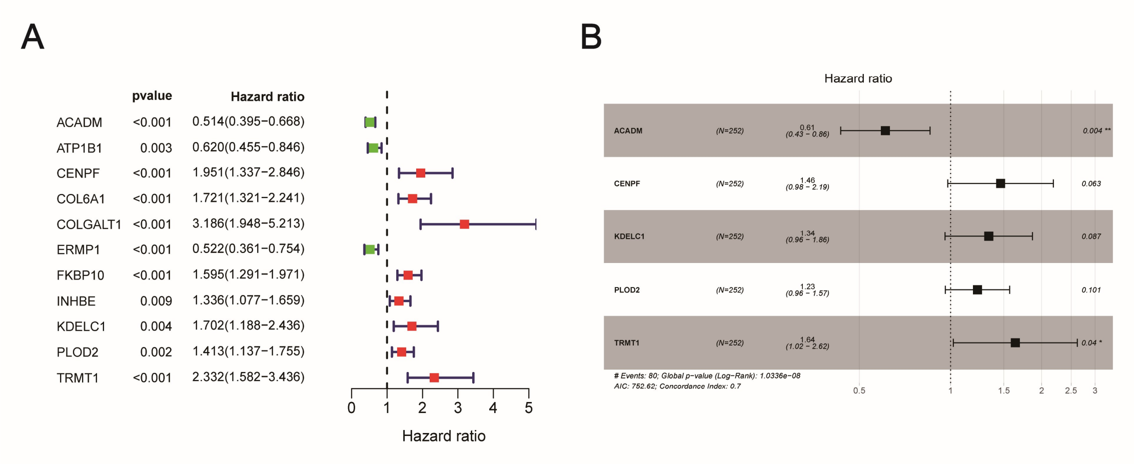

Figure S2. Identification of the candidate SARS-CoV-2 related genes in training set. (A) Univariate Cox regression analysis; (B) Multivariate Cox regression analysis to identify SARS-CoV-2 related genes correlated with OS.

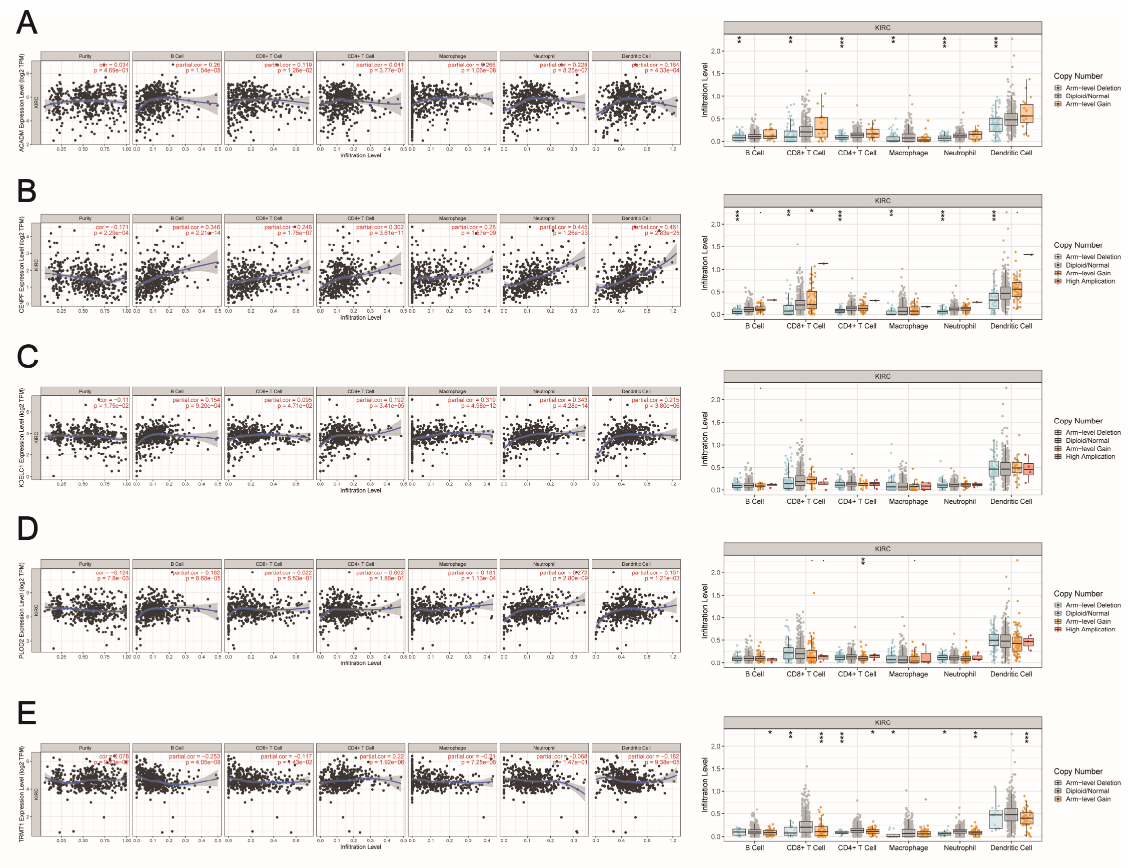

Figure S3. Correlation between the expression of ACADM, CENPF, KDELC1, PLOD2, TRMT1 and tumor purity or immune infiltration levels in KIRC through TIMER.

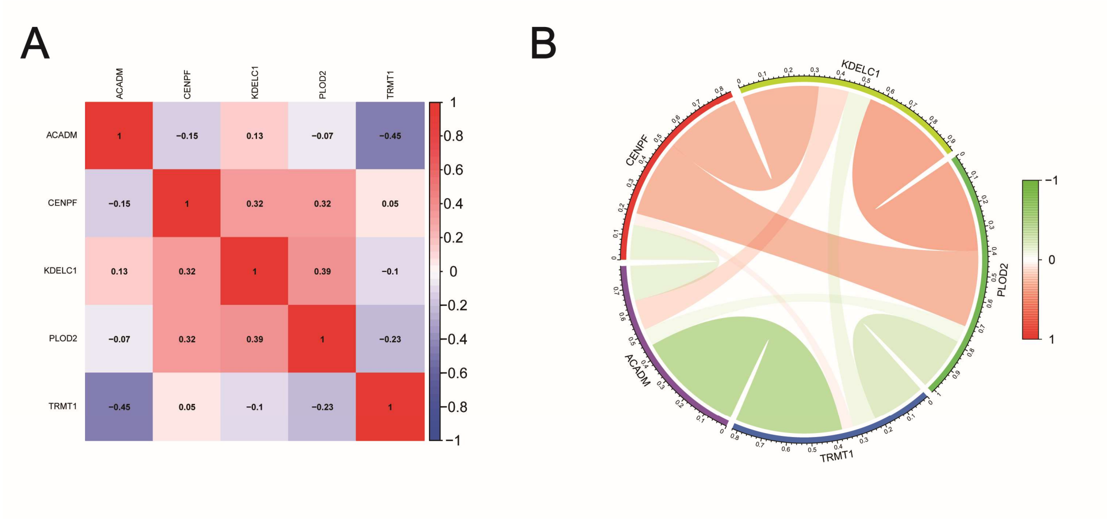

Figure S4. Correlation of the ACADM, CENPF, KDELC1, PLOD2, TRMT1 expression determined by Pearson correlation coefficient.
